# Somatic mutations reveal hyperactive Notch signaling in prurigo nodularis

**DOI:** 10.1172/jci.insight.172371

**Published:** 2026-04-22

**Authors:** Ahmad Rajeh, Shahin Shahsavari, Hannah Cornman, Alexander Kollhoff, Anuj Gupta, Mindy D. Szeto, Anusha Kambala, Olusola O. Oladipo, Varsha Parthasarathy, Junwen Deng, Melika Marani, Shirin Shahsavari, Selina M. Yossef, Vedha Vaddaraju, Waleed Adawi, Yagiz M. Akiska, Davies M. Gage, Sarah Wheelan, Thomas Pritchard, Madan M. Kwatra, Yevgeniy R. Semenov, Alexander Gusev, Won Jin Ho, Srinivasan Yegnasubramanian, Shawn G. Kwatra

**Affiliations:** 1 Department of Dermatology and; 2 Maryland Itch Center, University of Maryland School of Medicine, Baltimore, Maryland, USA.; 3 Sidney Kimmel Comprehensive Cancer Center, Johns Hopkins University, Baltimore, Maryland, USA.; 4 National Human Genome Research Institute, NIH, Bethesda, Maryland, USA.; 5 Department of Anesthesiology, Duke University School of Medicine, Durham, North Carolina, USA.; 6 Department of Dermatology, Massachusetts General Hospital, Boston, Massachusetts, USA.; 7 Department of Systems Biology, Harvard Medical School, Boston, Massachusetts, USA.; 8 Division of Genetics, Brigham & Women’s Hospital, Boston, Massachusetts, USA.; 9 Department of Medical Oncology, Dana-Farber Cancer Institute, Boston, Massachusetts, USA.; 10 Sidney Kimmel Comprehensive Cancer Center, Johns Hopkins University, Baltimore, Maryland, USA.; 11 Department of Oncology, Johns Hopkins University School of Medicine, Baltimore, Maryland, USA.

**Keywords:** Dermatology, Genetics, Genetic variation, Skin

## Abstract

Prurigo nodularis (PN) is a chronic inflammatory skin disease characterized by pruritic skin nodules of unknown etiology. Little is known about genetic changes in PN pathogenesis, particularly somatic events, which are often implicated in inflammatory conditions. We thus performed whole-exome sequencing on 54 lesional and nonlesional skin biopsies from 17 patients with PN and 10 patients with atopic dermatitis (AD) for comparison. Somatic mutational analysis revealed that PN lesional skin harbors recurrent somatic mutations in fibrotic, neurotropic, and cancer-associated genes that are absent in adjacent PN nonlesional skin. Nonsynonymous mutations were most frequently present in *NOTCH1* and the Notch signaling pathway, a key regulator of cellular proliferation and tissue fibrosis. In contrast, *NOTCH1* mutations were absent in AD. Somatic copy-number analysis, combined with expression data, identified recurrently deleted and downregulated genes in PN lesional skin, which are associated with axonal guidance and extension. Follow-up immunofluorescence validation demonstrated increased *NOTCH1* expression in PN lesional skin fibroblasts and increased Notch signaling in PN lesional dermis. Finally, a multicenter analysis revealed increased risk of *NOTCH1*-associated diseases in patients with PN. In characterizing the somatic landscape of PN, this study highlights the potential role of Notch pathway dysregulation in PN pathogenesis and fibrosis.

## Abstract 2

Extensive somatic mutations in prurigo nodularis lesions reveal new mechanistic insights into disease pathology.

## Introduction

Prurigo nodularis (PN) is a chronic inflammatory skin disease characterized by intensely pruritic, hyperkeratotic skin nodules on the trunk and extremities (1). Compared with more common and better-characterized chronic pruritic dermatoses like atopic dermatitis (AD) or psoriasis, PN is associated with greater itch intensity (2), as well as a marked quality of life impairment (3, 4). PN emerges in middle age, disproportionately affects African Americans, and is associated with multiple systemic conditions (5). Despite this substantial clinical burden, the etiology of PN remains poorly understood.

The current understanding of PN biology centers around an interplay between cutaneous inflammation, neuronal dysregulation, and altered keratinocyte differentiation and fibroblast signaling (6–8). Recent transcriptomic studies show characteristic patterns of immune polarization in patients with PN, including both Th2/Th17-centered cutaneous immune activation and cutaneous and systemic Th22-related cytokine upregulation (6). Distinct inflammatory signatures are seen in African American patients with PN, suggesting the existence of multiple disease endotypes (9, 10). However, whether these patterns are driven by genetic variation or environmental exposures remains unknown. In better-studied chronic pruritic dermatoses such as AD and psoriasis, genomic studies have accelerated our understanding of disease pathology and informed new treatments (11, 12); however, similar investigations are lacking for PN.

The relevance of genomic studies in inflammatory skin disease includes postzygotic variation. Somatic mutations throughout the body are known to drive neoplasms, but growing evidence also points to clonal expansions harboring somatic mutations in non-neoplastic disease and healthy-appearing tissue, including the skin (13–16). Such findings have informed our understanding of both the disease and the corresponding tissue biology. For example, colonic mucosa of patients with inflammatory bowel disease displays positive selection for mutations in IL-17 pathway genes, which may confer a protective advantage to mucosal epithelia (13, 17). Thus, delineating somatic events associated with PN may further our understanding of not only disease pathology, but also cutaneous molecular adaptations in the setting of chronic itch, fibrosis, and neuroinflammation. This is especially pressing given the comorbidities in patients with PN, including a higher risk of skin and internal malignancies (18, 19), which remain largely unexplained.

In this study, we characterize the landscape of somatic events in PN lesional skin. We perform whole-exome sequencing (WES) on individual-matched lesional and nonlesional skin biopsies from a diverse cohort of patients with PN, as well as patients with AD as a reference group. We explore the mutational landscape of PN at the individual nodule level, identifying somatic events in lesional PN skin compared to adjacent healthy-appearing skin. We also contrast the somatic profile of PN to that of AD to elucidate molecular pathways specific to PN. Our somatic analysis also leads us to functional and multicenter epidemiological follow-up investigations.

## Results

### Whole-exome analysis

**Figure 1:**
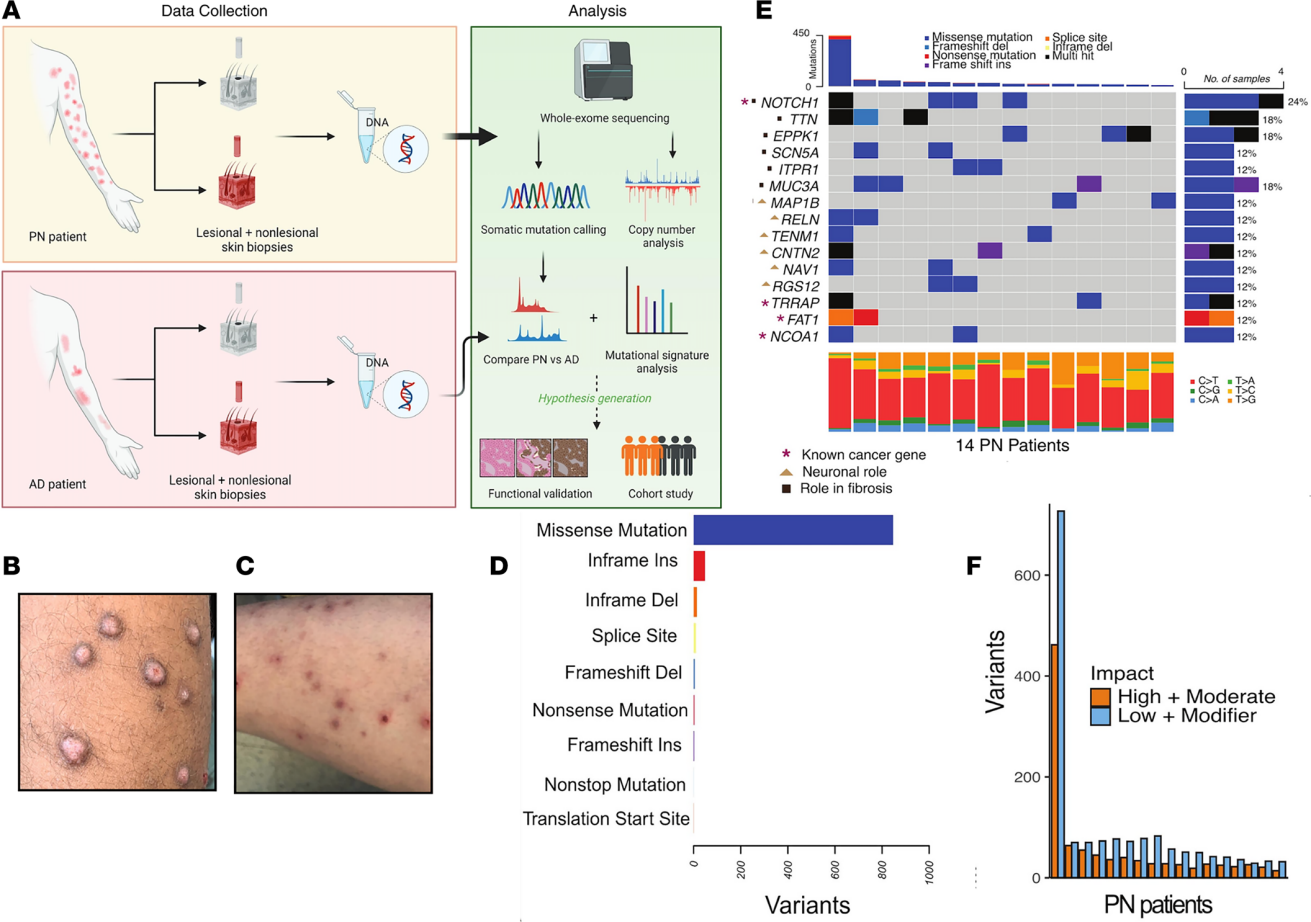
Somatic mutational landscape of PN. (**A**) Schematic of the study design, including sample collection and analysis. (**B** and **C**) Example skin images of 2 patients with prurigo nodularis (PN) enrolled in this study. (**B**) Dorsal left arm of an African American patient with scattered prurigo nodules. (**C**) Right arm of a White patient with scattered prurigo nodules. (**D**) Demographic information of patients with PN and atopic dermatitis (AD). (**E**) Waterfall plot displaying somatic mutations in 15 of the most frequently mutated genes in PN lesional skin compared with nonlesional skin. Ties were broken by known gene function. Columns represent the 17 patients with PN. Numbers shown on the right indicate the frequency of gene mutation. Total nonsynonymous somatic SNPs and indels per sample are displayed on top. One sample was hypermutated with 450 mutations. Bottom plot shows the frequency of different classes of SNVs. Variants annotated as “Multi hit” are those genes that mutated more than once in the same sample. (**F**) Total number of somatic mutations per sample, broken down by predicted impact on protein product based on snpEff, with functional (high or moderate) impact in yellow and nonfunctional (low or modifier) in blue.

A schematic overview of the study design is shown in Figure 1A. We recruited 17 patients with PN who met our selection criteria (see Methods) (Figure 1, B and C). Two skin punch biopsies were obtained from each patient: one from a prurigo nodule (lesional) and one from adjacent normal-appearing skin (nonlesional) within 10 cm of the lesion. WES was performed on 34 PN samples (lesional and nonlesional pairs from 17 patients with PN) and 20 samples from 10 patients with AD. One AD patient’s samples were removed due to low quality.

Across our PN cohort (34 WES datasets from 17 patients), we obtained approximately 1.7 billion reads, with an average sequencing depth of 195× (range: 166×–268×), covering more than 93% of the exome at a depth of 20× or greater. Similarly, in the AD cohort (18 WES datasets from 9 patients), sequencing yielded an average depth of approximately 187× (range: 156×–241×), with 94% of the exome covered at a depth of 20× or greater. One AD sample pair was excluded due to insufficient coverage in the nonlesional sample (38%). Detailed sequencing, alignment, and coverage metrics are summarized in Supplemental Table 1.

#### Somatic variation in PN lesional skin.

Somatic variants in PN nodules were identified by comparing PN lesional to nonlesional sample pairs of each patient. The landscape of somatic mutations in PN is shown in Figure 1, D–F. Following quality control, we identified 2,387 high-confidence somatic single nucleotide variations (SNVs) and small insertions/deletions (indels) affecting 1,933 genes from PN lesional skin. Mutational analysis showed a median of 75 lesion-specific somatic mutations per patient, with a median mutational burden of approximately 0.52 per megabase (range: 0.26 to 9 per megabase; an outlier sample was further characterized in Supplemental Figure 1; supplemental material available online with this article; https://doi.org/10.1172/jci.insight.172371DS1). Among these, 35% were missense (847 of 2,387), 2.0% nonsense (48 of 2,387), 0.59% splice-site altering (14 of 2,387), 0.58% in-frame indels (13 of 2,387), and 0.3% frameshift indels (8 of 2,387). Based on predicted functional impact, variants were categorized as “High + Moderate,” indicating likely change in protein function, or “Low + Modifier,” indicating minimal impact in protein function (Figure 1F). Each PN sample harbored a median of 29 functionally lesion-specific somatic SNVs or indels (range: 13 to 450) (Figure 1F).

**Figure 2:**
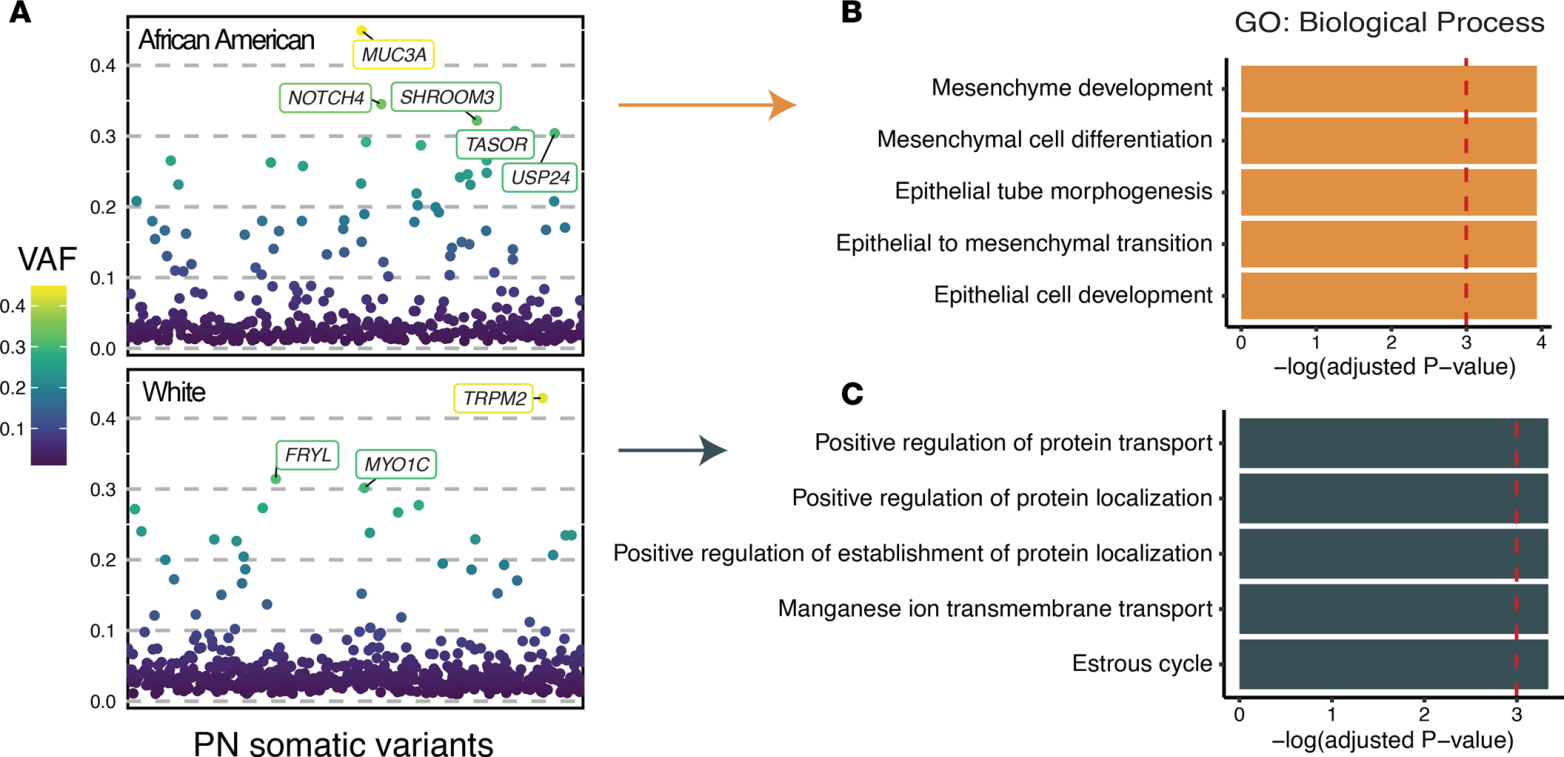
Distinct somatic selection in lesional skin of African American patients with PN. (**A**) Nonsynonymous somatic mutations are ordered by their genomic locations on the *x*-axis and the corresponding VAF is shown on the *y*-axis. Variants with 0.3 or higher VAF are labeled with their gene name. (**B** and **C**) Bar graphs showing the pathway enrichment results of high-VAF (>0.3) nonsynonymous mutations in African American and White patients with PN, respectively. Hypergeometric test with Benjamini-Hochberg FDR correction (FDR < 0.05).

**Figure 3:**
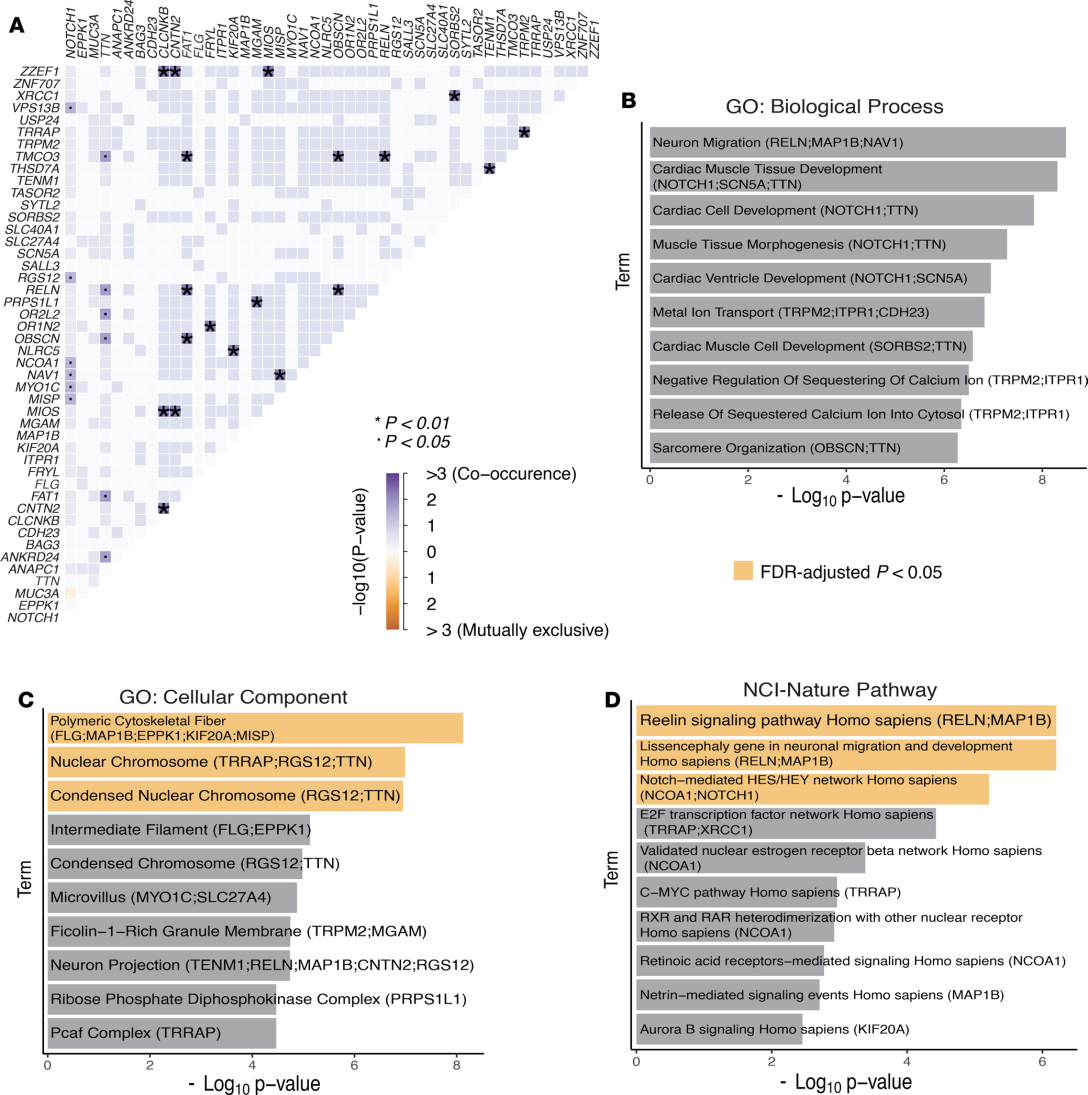
Co-occurrence and enrichment of recurrent somatic mutations in PN lesional skin. (**A**) Somatic mutational correlation matrix of all 46 genes with recurrent nonsynonymous somatic mutations in PN lesional skin. Significant pairs were identified with Fisher’s exact test. (**B**–**D**) Gene ontology (GO) term and pathway enrichment results of recurrently mutated genes across 3 pathway databases. Terms with FDR-corrected *P* < 0.05 are colored in yellow. Fisher’s exact test was used for co-occurrence and hypergeometric enrichment testing with Benjamini-Hochberg FDR correction (FDR < 0.05).

The median somatic variant allele frequency (VAF) in PN lesional skin was 0.031 (range: 0.01 to 0.449), with White patients with PN displaying a higher VAF than African American patients with PN (0.033 compared to 0.029, *P* = 0.0056, Wilcoxon’s test) (Figure 2A). At the gene level, there were 46 genes with nonsynonymous somatic mutations in at least 2 out of 17 patients with PN. These recurrently mutated genes were categorized into 3 predefined biological groups: 5 genes associated with pathologic fibrosis (*NOTCH1*, *SCN5A*, *MAP1B*, *TTN*, and *ITPR1*) (20), 6 genes involved in neuronal migration or projection (*MAP1B*, *RELN*, *TENM1*, *CNTN2*, *NAV1*, and *RGS12*), and 4 cancer-associated genes (*NOTCH1*, *TRRAP*, *FAT1*, and *NCOA1*), according to the Cancer Gene Census (CGC) from the Catalogue of Somatic Mutations in Cancer (COSMIC) (21). *NOTCH1* was the most frequently mutated gene, with 4 missense SNVs (p.Arg1962His, p.Asn70Ile, p.Glu450Lys, and p.Asn325Lys) and 1 in-frame deletion (p.Val413-Asp414del) detected across 4 patients. *NOTCH1* is an intracellular regulator of the Notch family with pleiotropic functions in cellular proliferation and tissue fibrosis, and is a well-established driver of cutaneous squamous cell carcinoma (cSCC) (22, 23). Three out of 5 identified *NOTCH1* mutations were within the extracellular epidermal growth factor–like (EGF-like) domain, which is involved in ligand binding and prevention of constitutional activation (24). Somatic mutations in *NOTCH1* co-occurred with mutations in *NCOA1*, *MISP*, *NAV1*, *MYO1C*, *RGS12*, and *VPS13B* (*P* < 0.05, pairwise Fisher’s exact test) (Figure 3A).

#### Fibrosis-associated genes.

In addition to *NOTCH1*, 2 missense mutations in *SCN5A* (p.Gly1158Asp and p.Arg1638Gln) were identified across 2 patients with PN. *SCN5A* encodes the α subunit of the Na_v_1.5 sodium channel, which is essential for cardiomyocyte depolarization and is linked to cardiac fibrosis (25). A member of the same sodium channel family, *SCN10A*, encodes Na_v_1.8 and is associated with cutaneous neuroinflammation. *SCN10A* is upregulated in the epidermis of rosacea and psoriasis lesions (26).

Two missense mutations in *ITPR1* (p.Ala805Val and p.Glu914Lys) were identified in 2 patients with PN. *ITPR1* encodes 1 of the 3 members of the IP_3_ receptor, which forms calcium channels and is associated with pancreatic fibrosis (20), in addition to chronic itch mediated by astrocytes (27). Two recurrent missense mutations in *MAP1B* (p.Val1549Gly × 2) were also identified in 2 patients with PN. While *MAP1B* is classically associated with neurogenesis and microtubule assembly, it has also been implicated in fibrosis, particularly in the eye (20, 28).

Mutations were also detected in *TTN*, a gene with a well-established role in interstitial fibrosis. TTN was mutated in 3 patients, with 4 missense mutations (p.Ala25524Val, p.Asn8023Lys, p.Ala35193Thr, and p.Val7022Ala) and 1 frameshift deletion (p.Val29546AsnfsTer3). *TTN*, the largest protein-coding gene in humans, has an established role in interstitial fibrosis and is frequently mutated in numerous cancers (29, 30). Additionally, a recurrent missense mutation in *EPPK1* (p.Ile1690Val) was identified in 3 patients with PN. While *EPPK1* was not in our predefined gene sets, it was the only gene with 3 or more identical recurrent somatic mutations, at either the DNA or amino acid level. *EPPK1* encodes a plakin family protein that is involved in cytoskeletal organization and has been shown to accelerate keratinocyte migration during wound healing (31, 32). Given the chronic skin remodeling and fibrosis seen in PN, *EPPK1* dysregulation may contribute to epidermal hyperplasia and fibrosis.

#### Neurotropic genes.

*MAP1B*, previously noted for its involvement in fibrosis, also functions in microtubule assembly and axonal growth (20, 28). *RELN*, a key regulator of neuronal migration and synaptic plasticity, harbored 2 missense mutations (p.Thr468Pro and p.Val167Leu) in 2 patients. Missense mutations were also found in *TENM1* (p.Ala2661Ser and p.Gly1134Arg) and *CNTN2* (p.Ala2661Ser and p.Gly1134Arg) across 2 patients. *TENM1* encodes a protein of the teneurin subfamily, which is thought to function as a neuronal cellular signal transducer and is among the most highly mutated genes in melanoma (33). *CNTN2*, which encodes contactin 2, plays a role in neuronal excitability (34). In addition, 2 missense mutations were found in *RGS12* (p.Gly454Asp and p.Arg403Cys) and *NAV1* (p.Pro1507Ser and p.Arg1581Cys) across 2 patients. *RGS12* is thought to be a critical modulator of serotonergic neurotransmission (35), while *NAV1* is a relatively understudied gene that is involved in neuronal development and regeneration (36).

#### Cancer-associated genes.

Our set of 46 recurrently mutated genes with nonsynonymous mutations was compared to the curated CGC gene list from COSMIC (21). This analysis revealed recurrent mutations in *TRRAP*, *FAT1*, and *NCOA1*. *TRRAP*, a key regulator of cell cycle, harbored 3 mutations (p.Pro252Ser, p.Pro1364Leu, and p.Glu2479Lys) across 2 patients with PN. *TRRAP* is frequently mutated in melanoma and alters fibroblast gene expression that affects neuronal function and ion transport (37, 38). *FAT1*, a regulator of cell-cell adhesion and extracellular matrix integrity and a known driver of cSCC (39), had a nonsense mutation (p.Gln2076Ter) and a splice site mutation in 2 patients. Additionally, *NCOA1* harbored 2 missense mutations (p.Pro1102Ser and p.Ala498Thr) across 2 patients with PN.

Oncogenic pathways in PN were further examined using curated datasets from The Cancer Genome Atlas (TCGA) (40). The Notch signaling pathway was the most frequently mutated, with variants detected in 9 out of 17 patients (52.9%), including mutations in *NOTCH1*, *NOTCH4*, *CNTN6*, *FBXW7*, *JAG2*, and *SPEN*. The RTK-RAS and Wnt pathways were also affected, with mutations present in 5 (29.4%) and 4 (23.5%) patients with PN, respectively. A list of high-confidence somatic SNVs and indels identified in this study is listed in Supplemental Table 2.

#### Gene set enrichment analysis.

Gene set enrichment analysis (GSEA) provides an unbiased pathway-level somatic landscape, highlighting patterns that are too subtle to detect at the gene level (41). To assess somatic selection, an enrichment analysis of high-VAF (>0.3) somatic mutations was conducted, revealing distinct racial patterns (Figure 2A). Of note, high-VAF mutations in African American patients with PN were associated with 2 epithelial-mesenchymal transition genes, *NOTCH4* and *TASOR* (FDR-adjusted *P* < 0.05).

Subsequently, a broader enrichment analysis of all recurrently mutated genes in lesional PN was performed using 3 term databases: Gene Ontology (GO) Biological Process, GO Cellular Component, and NCI-Nature Pathways. After correction for multiple hypothesis testing, the most commonly mutated pathways included Notch-mediated signaling, neuronal migration, and polymeric cytoskeletal fiber organization (Figure 2, B and C).

To further investigate relationships among recurrent somatic changes, we assessed co-occurrence and pathway enrichment across the 46 recurrently mutated genes. Several fibrosis-, neuron- and cancer-associated mutations showed significant co-occurrence, and functional annotation highlighted enrichment in Notch-mediated signaling and axonal guidance pathways. These findings emphasize the convergence of somatic events on fibrotic and neurotropic processes in PN lesional skin (Figure 3, A–D).

**Figure 4:**
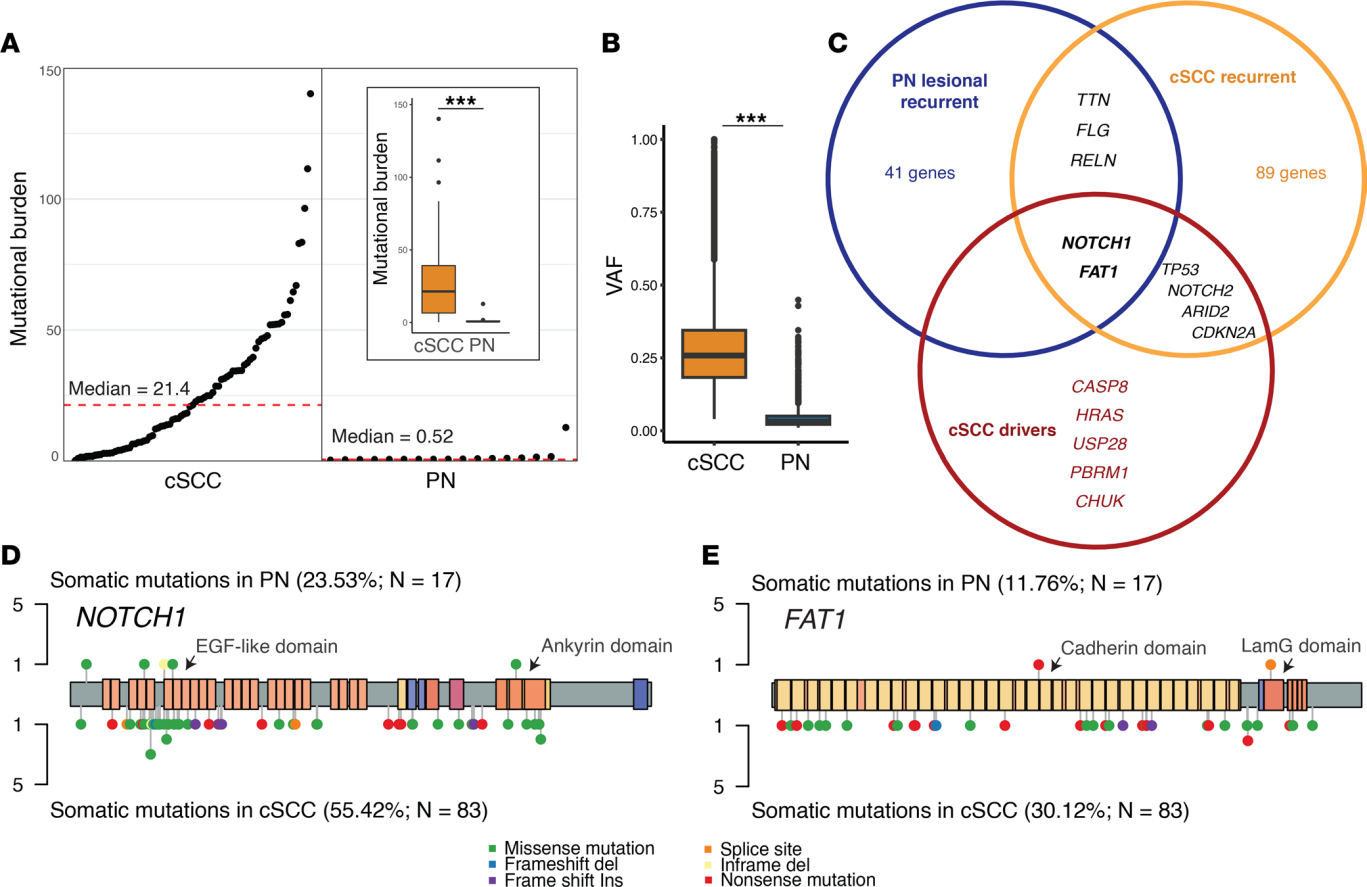
Somatic mutations in PN and cSCC. (**A**) Rank order plot showing the somatic mutational burden (number of nonsynonymous mutations occurring per megabase of coding regions) across 17 patients with PN and 83 patients with cutaneous squamous cell carcinoma (cSCC). Red dashed line indicates the median mutational burden. Inset is a box-and-whisker plot comparison of somatic mutational burden between the 2 cohorts. (**B**) Box-and-whisker plot showing VAF of all nonsynonymous somatic variants in patients with cSCC and PN. (**C**) Venn diagram depicting the overlap between known cSCC driver genes, genes that were found mutated in 25% or more of the 83 cSCC samples (cSCC recurrent), and genes that were found mutated in 2 or more of our 17 PN samples (PN lesional recurrent). (**D** and **E**) Somatic mutations falling within the *NOTCH1* and *FAT1*, respectively. The *y*-axis indicates the number of patients with PN (top) or cSCC (bottom) the carrying the somatic mutation. Colored rectangles indicate known functional domains of the protein product. EGF, epidermal growth factor; LamG, laminin G. PN versus cSCC mutational burden and VAF were compared using 2-sided Mann-Whitney *U* tests.

Since *NOTCH1* was the most frequently mutated gene in our PN lesional skin cohort and loss-of-function mutations in *NOTCH1* have been well established in cSCC (42), a comparison was performed between our PN somatic variants and a publicly available cSCC variant dataset from a meta-analysis by Chang et al. (39). Among 83 cSCC samples, the median tumor mutational burden was 21.4, and the median VAF was 0.258, both significantly higher than in PN samples (*P* < 0.001, Wilcoxon’s test) (Figure 4, A and B). *NOTCH1* was mutated in 55.4% of cSCC samples, second only to *TTN*. Overall, 6 genes were mutated in at least 25% of patients with cSCC and in 2 or more of our patients with PN: *NOTCH1*, *FAT1*, *TTN*, *FLG*, and *RELN* (Figure 4, C–E). Of those genes, *NOTCH1* and *FAT1* have been previously identified as likely cSCC drivers (39). Additionally, *TASOR2*, a gene differentially regulated in several cancers (43), was the only gene not mutated in any of the cSCC samples while having somatic mutations in 2 of our patients with PN.

### Somatic copy number variation in PN

**Figure 5:**
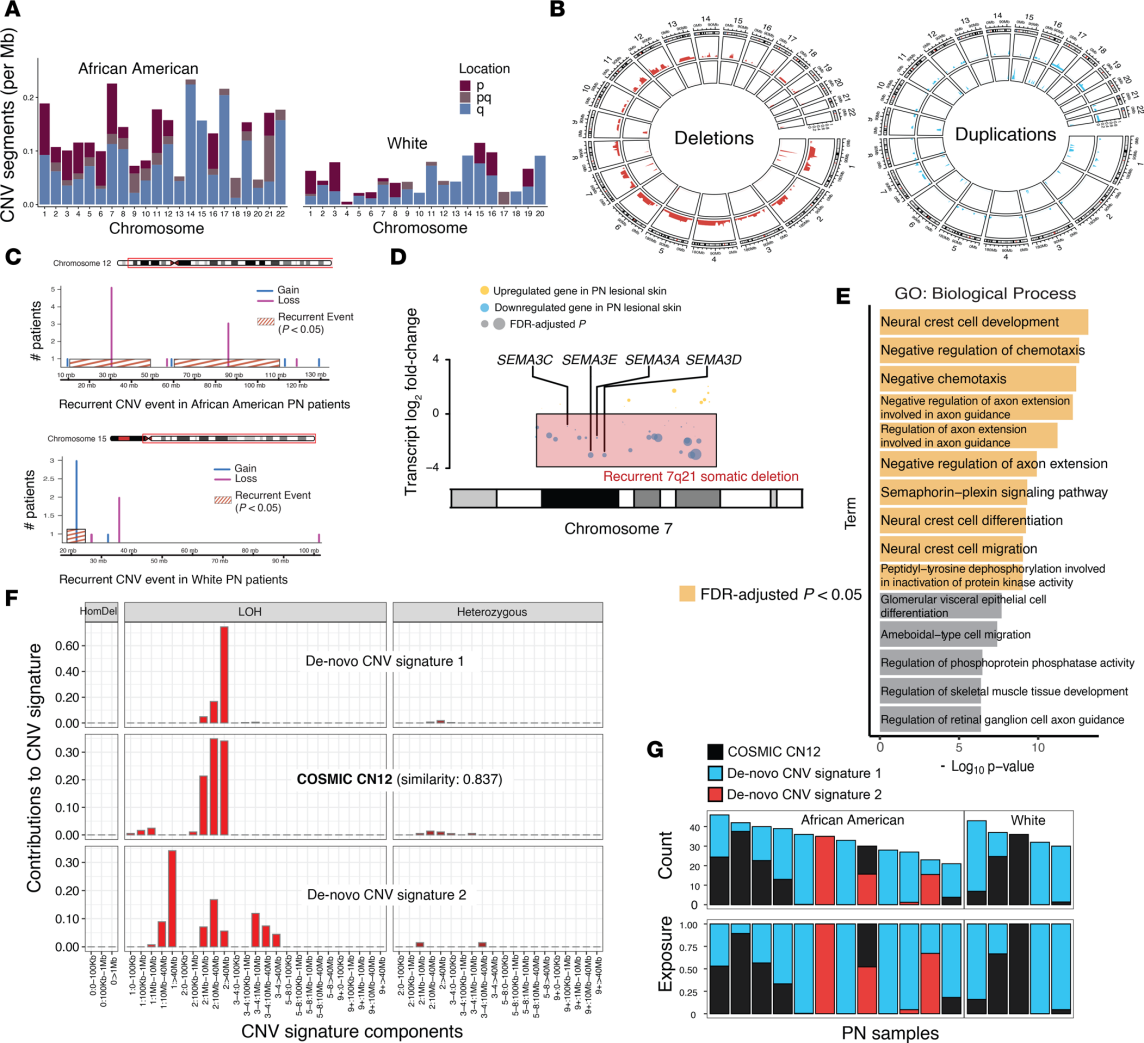
Landscape of somatic CNVs in PN. (**A**) Normalized copy number per sample per chromosome for African American and White patients. (**B**) Circular plot illustrating the proportion of samples with deletions (red) and duplications (blue) per genomic region for African American patients (outer ideogram) and White patients (inner ideogram). (**C**) Examples of recurrent somatic CNV events only observed in African American patients or White patients on chromosomes 12 and 15, respectively. (**D**) RNA-seq data were used to determine differentially down- and upregulated genes in PN lesional skin. Transcript log_2_(fold change) is shown on the *y*-axis with respect to genomic location on chromosome 7 on the *x*-axis. Overlayed red box shows the location of the recurrent 7q21 somatic deletion. (**E**) GO term enrichment results of 264 genes that overlapped recurrent deletions while also being differentially downregulated in PN lesional skin. Terms with FDR-corrected *P* < 0.05 are colored in yellow. (**F**) Decomposition plot showing the relative contributions of CNV signature components to the 3 somatic CNV signatures detected. HomDel, homozygous deletion; LOH, loss-of-heterozygosity. (**G**) Distribution of CNV signatures in African American and White patients with PN. Differential expression was assessed using the DESeq2 Wald test with Benjamini-Hochberg FDR correction (adjusted *P* < 0.05). GO analyses were performed using hypergeometric testing with FDR correction.

In addition to somatic SNVs and indels, somatic copy number variations (CNVs) may contribute to PN pathogenesis by altering gene dosage. Copy number analysis identified a median of 66 somatic CNVs per PN patient (range: 48 to 344). Recurrent deletions were detected in chromosomes 1p13 (6/17; 35%), 4p (5/17; 29%), 4q13.2 (4/17; 24%), 5q (3/17; 18%), 7q21 (3/17; 18%), 11q14 (5/17; 29%), 12 (7/17; 41%), and 19q13.42 (4/17; 24%). Additionally, significant copy-number gains were observed in chromosomes 7p22 and 17q25.3 in at least one patient (*P* < 0.05; see Methods). Distinct racial pattern CNVs were noted; White patients had more deletions in chromosome 1p13.3 (4/5; 80%) and duplications in chromosome 15 (3/5; 60%), whereas African American patients exhibited deletions in chromosomes 4p (5/12; 42%), 4q13.2 (4/12; 33%), 7q21 (3/12; 25%), 11q14 (5/12; 42%), and 12 (5/12; 42%) (Figure 5, A–C). Overall, 3,173 gene loci overlapped with recurrent deletions, while 5 gene loci were affected by recurrent duplications: *FOXK2*, *WDR45B*, *EIF3B*, *RSPH10B2*, and *CCZ1B*.

CNVs are known modulators of gene expression and biological processes (44, 45). To assess their functional impact in PN, we overlapped recurrent CNV loci with differential expression results from PN lesional skin RNA-seq data (6). A recurrent 7q21 deletion encompassing *SEMA3C*, *SEMA3E*, *SEMA3A*, and *SEMA3D* accounted for much of the signal observed (Figure 5D). In total, 264 significantly downregulated genes (FDR-adjusted *P* < 0.05; log[fold change] < 0) overlapped with recurrent deletions in PN lesions, with pathway enrichment showing strong associations with neural crest cell development, negative regulation of chemotaxis, and negative regulation of axonal extension (Figure 5E). Two loci, *FOXK2* and *EIF3B*, were recurrently duplicated and correspondingly upregulated in PN lesional skin. Notably, repeating the enrichment analysis using expression data alone did not identify neuronal pathways (Supplemental Figure 2), underscoring the specific contribution of CNVs to neurotropic pathway dysregulation in PN.

Finally, CNV signature analysis was conducted to investigate the common etiology of these somatic events. Three CNV signatures were identified in our PN samples based on loss-of-heterozygosity status, total copy number state, and segment length. One CNV signature showed high similarity (0.837 cosine similarity) to CN12 from the COSMIC curated database, which is believed to indicate chromosomal instability associated with genome doubling events (46). African American and White patients displayed similar exposure to CN12 (*P* = 0.58, Fisher’s exact test) (Figure 5, F and G).

### Somatic mutational landscape of PN compared to AD

**Figure 6:**
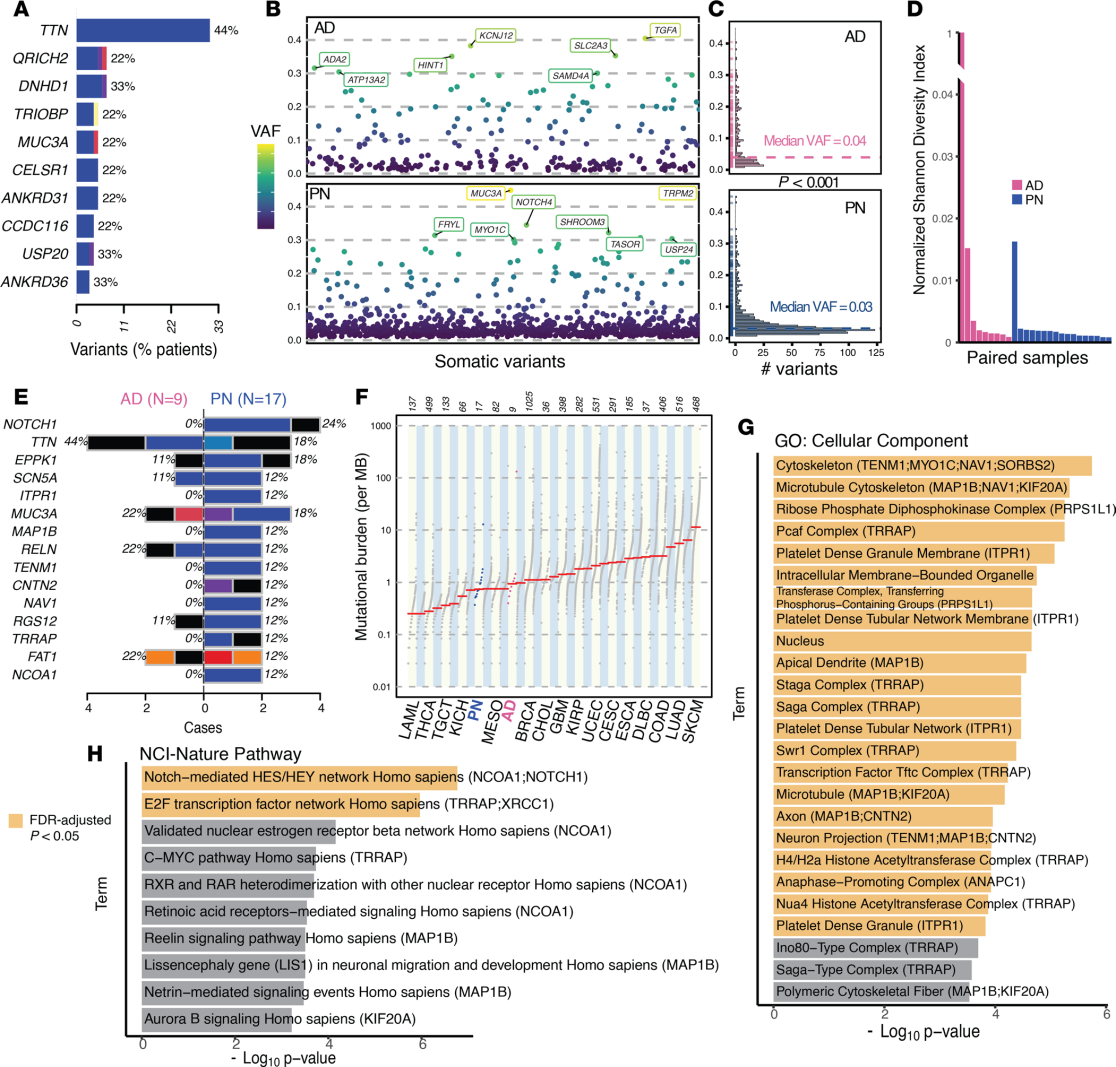
Somatic mutational differences between PN and AD. (**A**) Ten genes with the most frequent nonsynonymous somatic mutations in our AD cohort. (**B**) All nonsynonymous somatic mutations in AD and PN are ordered by their genomic locations on the *x*-axis and the corresponding VAF is shown on the *y*-axis. Variants with 0.3 or higher VAF are labeled with their gene name. (**C**) Histograms showing the frequency of nonsynonymous somatic variants on the *x*-axis at the corresponding VAF on the *y*-axis, in AD and PN. *P* value indicates the significant difference in means (Wilcoxon’s test). (**D**) Mutational diversity per sample using a normalized Shannon diversity index based on the VAF of somatic SNVs and indels in patients with PN and AD. One hypermutated AD sample was not excluded (see Methods). (**E**) The top mutated genes in our PN cohort are illustrated, with the corresponding relative frequency and percentage of alteration in AD and PN samples. (**F**) Rank order plot showing the somatic mutational burden per megabase across 17 patients with PN, 9 patients with AD, and all 33 cancers in The Cancer Genome Atlas cohort. Red lines indicate the median mutational burden. A dictionary for cancer symbols is included in Supplemental Table 3. (**G** and **H**) GO term enrichment results of 21 genes with nonsynonymous somatic mutations in at least 2 PN samples and no AD samples across 2 pathway databases. Terms with FDR-corrected *P* < 0.05 are colored in yellow. PN versus AD VAF and Shannon diversity were compared using Mann-Whitney *U* tests. Mutational burden across PN, AD, and TCGA cancers was compared using a Kruskal-Wallis test. GO enrichment analyses were performed using hypergeometric testing with FDR correction.

Somatic mutational analysis of AD lesional skin revealed a median of 75 lesion-specific mutations per patient (range: 37 to 1161). The most frequently mutated genes in AD lesional skin included *TTN* (4/9; 44%), *DNHD1* (3/9; 33%), *USP20* (3/9; 33%), and *ANKRD36* (3/9; 33%) (Figure 6A). AD lesional skin had a significantly higher median somatic mutational burden (0.66 per megabase; range: 0.28 to 92.7) and VAF (median: 0.04; range: 0.010 to 0.405), compared with PN (*P* < 0.001, Wilcoxon’s test) (Figure 6, B–F). Of note, none of the AD samples had somatic mutations in *NOTCH1* (Figure 6E).

#### Distinct mutations in PN.

To identify PN-specific mutational patterns, we compared the 46 genes recurrently mutated in PN to those in AD. This analysis identified 21 genes uniquely altered in PN, with enrichment in Notch-mediated HES/HEY signaling, E2F transcription factor activity, and neuronal migration pathways, suggesting potential mechanistic links between somatic mutations and PN’s fibrotic and neuroinflammatory phenotype (Figure 6, G and H).

#### Shared somatic mutations between PN and AD.

Among the shared mutations, *DUOX2*, a gene involved in hydrogen peroxide release through NADPH oxidase, harbored 1 nonsense mutation (p.Trp1374Ter) and 2 missense mutations (p.Glu1017Gly and p.Ser1067Leu) in 2 patients with AD. The same nonsense mutation in *DUOX2* appeared in 1 PN sample, making it the only shared mutation between both conditions. *DUOX2* variants have been associated with elevated plasma IL-17C levels, a cytokine associated with chronic inflammation and epithelial barrier dysfunction (47, 48).

#### Mutational signatures.

**Figure 7:**
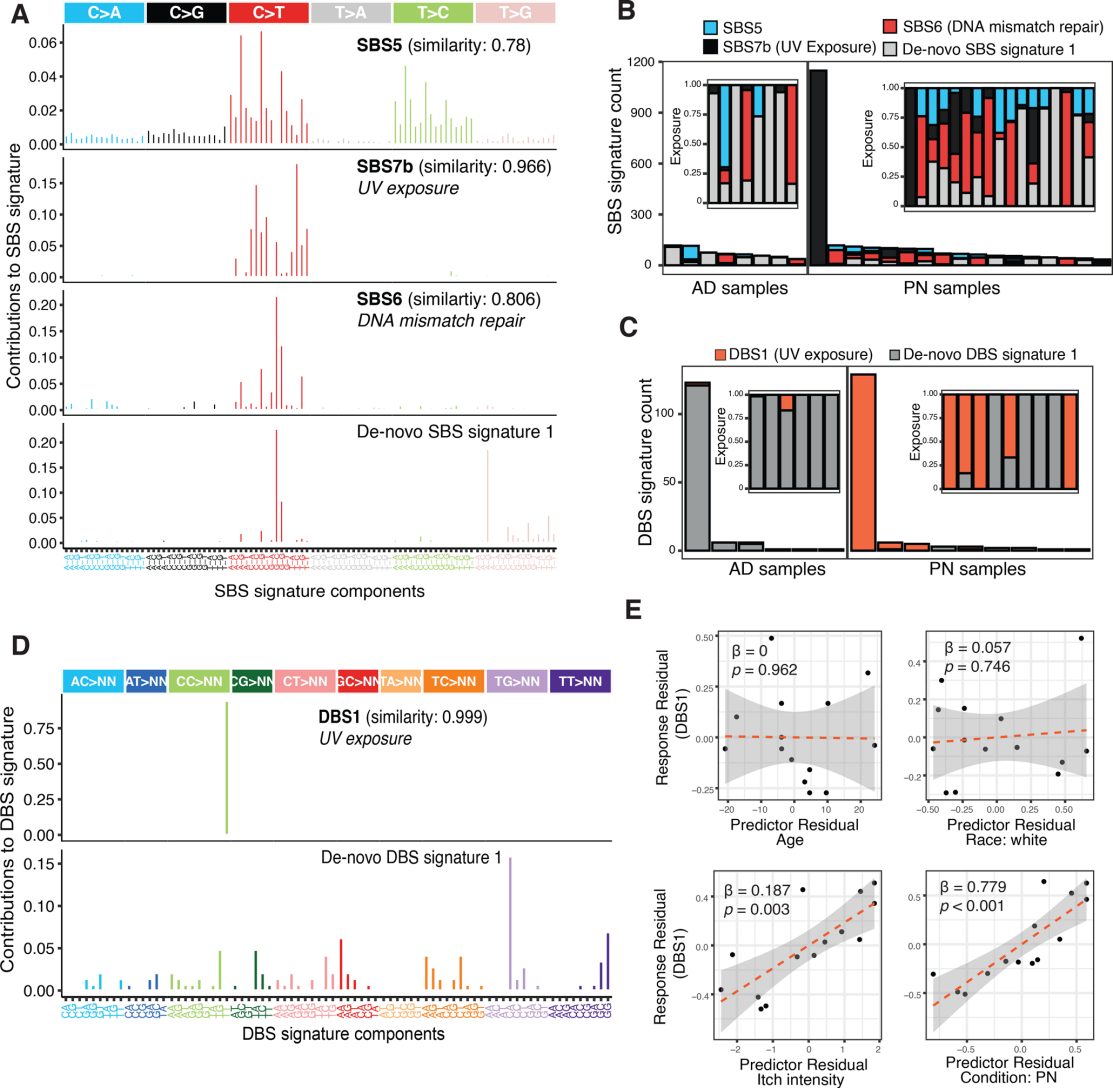
Somatic mutational signatures in PN and AD. (**A**) Decomposition plots of the 4 somatic SBS signatures detected showing the relative proportion of each transition and transversion subtype. (**B**) Distribution of SBS signatures in PN and AD samples. The inset figure shows the relative exposures of mutational signature types. Not shown, but included in all analyses, is a hypermutated AD sample with >20,000 instances of SBS5. (**C**) Box-and-whisker plots showing the distribution of SBS signatures in PN compared to AD. (**D**) Decomposition plot of the 2 somatic DBS signatures detected showing the relative proportion of each base-pair mutation subtype. (**E**) Distribution of DBS signatures in PN and AD samples. The inset figure shows the relative exposures of DBS signature types. Samples not shown did not display any known DBS signatures. (**E**) Partial regression plots of DBS1 on each of age, race, itch intensity, and condition (PN versus AD), after controlling for the remaining covariates. Overall model-adjusted *R*^2^ = 0.66. PN versus AD SBS and DBS signature exposures were compared using Mann-Whitney *U* tests. DBS1 associations were evaluated using a multivariable linear regression model adjusting for age, sex, race, itch intensity, and diagnosis.

Somatic mutational signature analysis identified 4 single base substitution (SBS) signatures across PN and AD samples, including SBS7b (UV-related; cosine similarity 0.966), SBS6 (mismatch repair–related; similarity 0.806), and SBS5 (clock-like; similarity 0.781) based on COSMIC reference signatures (49), along with a de novo PN/AD-associated SBS signature enriched for A[T>]G and G[C>T]C substitutions (Figure 7, A–C). One hypermutated AD sample (>20,000 SBS5 events) was excluded from visualization. Analysis of doublet base substitutions identified 2 double base substitution (DBS) signatures: COSMIC-matched DBS1 (similarity 0.999), characterized by increased CC>TT substitutions with transcriptional strand bias, and a de novo DBS signature enriched for TG>CA substitutions (Figure 7, D and E). In a multivariable regression model adjusting for age, sex, race, itch intensity, and condition (PN versus AD), PN diagnosis (*P* < 0.001) and itch intensity (*P* = 0.003) were independently associated with DBS1 exposure (adjusted *R*^2^ = 0.66, model *P* = 0.004) (Figure 7E).

#### Immunofluorescence analysis.

**Figure 8:**
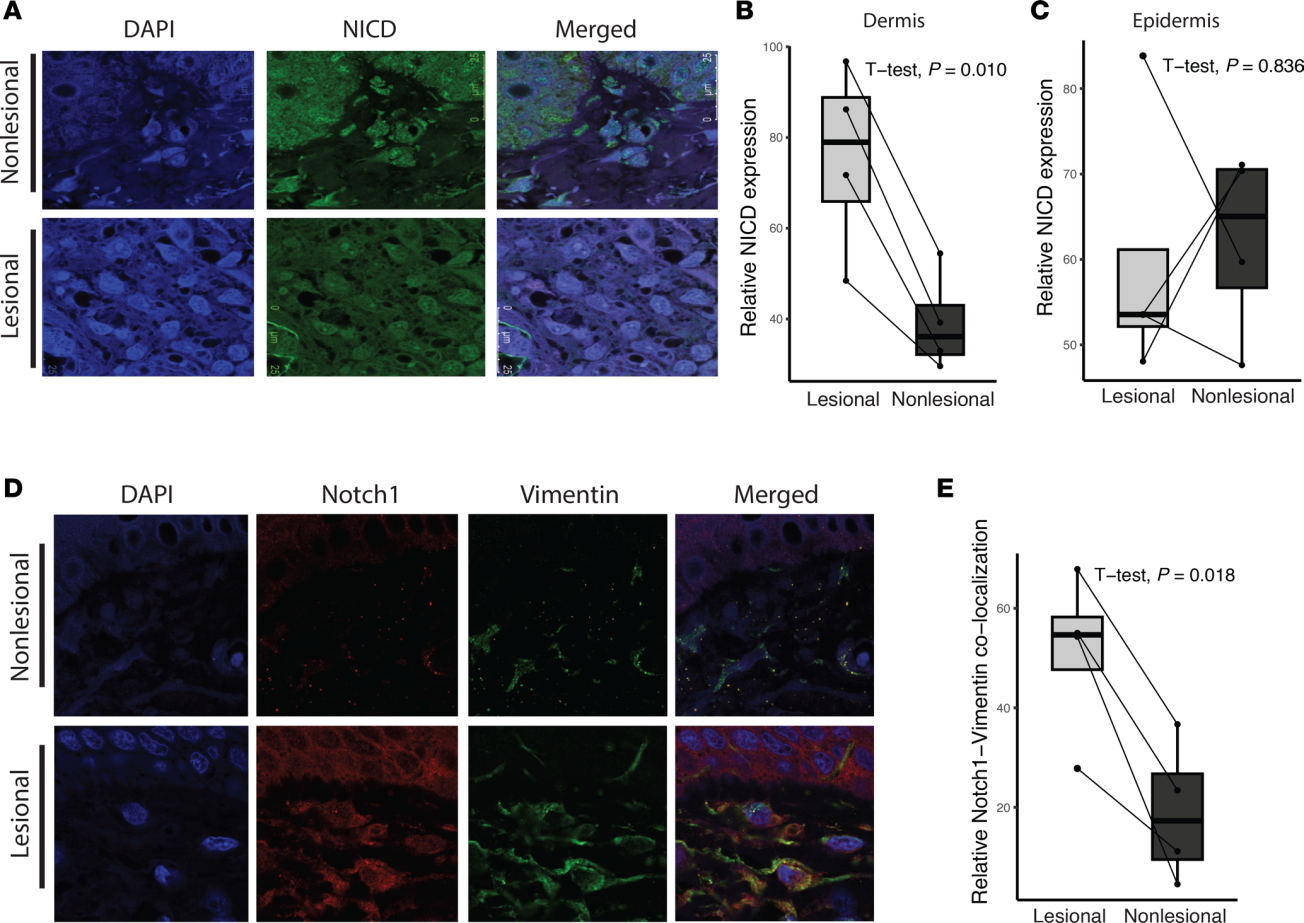
Notch signaling is activated in lesional skin of patients with PN. (**A**) IF staining of NICD in lesional and nonlesional skin sections of a patient with PN with a NOTCH1 somatic mutation. Representative skin sections are shown at 189× magnification of the dermis. NICD (green), DAPI (blue). (**B** and **C**) Paired box-and-whisker plots showing the difference in relative expression of NICD between lesional and nonlesional PN dermis (**B**) and epidermis (**C**). (**D**) IF staining of NOTCH1 and vimentin in lesional and nonlesional skin sections of a patient with PN. NOTCH1 (red), vimentin (green), DAPI (blue). Representative skin sections are shown at 189× magnification of the dermis. (**E**) Paired box-and-whisker plot showing the difference in relative colocalization of NOTCH1 and vimentin between PN lesional and nonlesional skin. Lesional versus nonlesional comparisons were performed using paired Wilcoxon’s signed-rank tests.

To functionally validate *NOTCH1* mutations detected in PN lesional skin, immunofluorescent (IF) staining was performed on the 4 lesional and nonlesional PN sample pairs with confirmed *NOTCH1* mutations (Figure 8A). Staining for the Notch intracellular domain (NICD), a marker of active Notch signaling, was observed in both lesional and nonlesional skin of PN skin. However, NICD immunofluorescence signal was significantly higher in lesional dermis compared with the nonlesional dermis from the same patients (*t*[3] = 5.92, *P* = 0.010, paired Student’s *t* test). No significant difference was observed in NICD immunofluorescence between lesional and nonlesional PN epidermis (*t*[3] = –0.226, *P* = 0.836) (Figure 8, B and C). Further analysis revealed significantly increased colocalization of NOTCH1 with vimentin, a fibroblast marker, in lesional PN skin compared with nonlesional skin of the same patients (*t*[3] = 4.77, *P* = 0.018) (Figure 8, D and E). While NOTCH1 also colocalized with KRT10, a keratinocyte marker, there was no significant difference in colocalization between lesional and nonlesional PN skin (*t*[3] = –2.09), *P* = 0.128) (Supplemental Figure 3).

#### Multicenter analysis.

**Figure 9:**
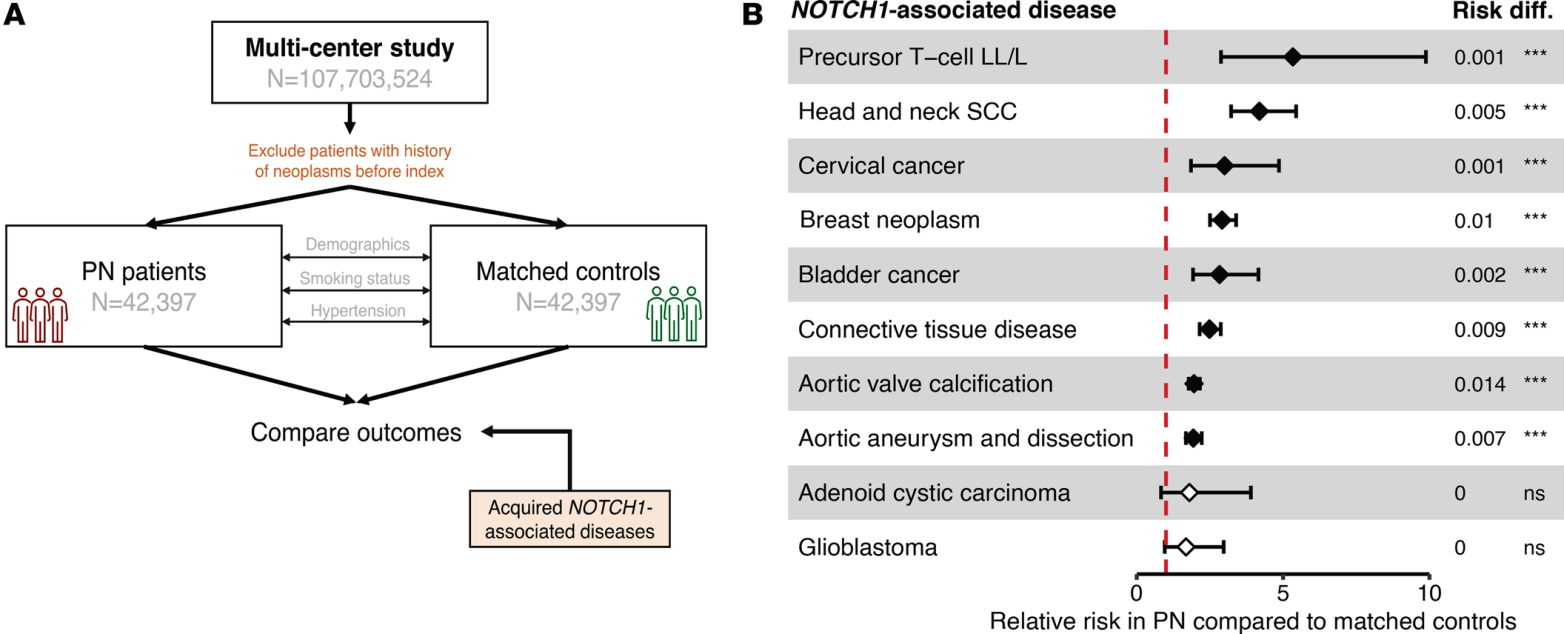
Higher risk of NOTCH1-associated diseases in patients with PN. (**A**) A multicenter cohort of patients with PN and propensity-score-matched controls was obtained using the TriNetX Research Network. The top 10 acquired, nonredundant diseases associated with NOTCH1 were determined based on available literature support using the DisGeNET database. (**B**) Cumulative relative risk of NOTCH1-associated diseases in patients with PN compared to matched controls. LL/L, lymphoblastic leukemia/lymphoma; H&N SCC, head and neck squamous cell carcinoma. ***P < 0.0001. NS, no significant difference in risk. Relative risk estimates were calculated using the TriNetX analytics platform following 1:1 propensity score matching; *P* < 0.05 was considered significant.

To assess the clinical relevance of our findings for patients with PN, we first identified the top 10 noncongenital, nonredundant diseases with the highest evidence for *NOTCH1* involvement, as determined by the gene-disease association (GDA) score from DisGeNET (50). The highest GDA scores included aortic valve calcification (GDA 0.65), precursor T cell lymphoblastic leukemia/lymphoma (GDA 0.6), and head and neck SCC (GDA 0.6). We then leveraged a multicenter cohort through the TriNetX Research Network. A total of 42,397 PN patients without a history of any neoplasms were identified. Controls were identified through 1:1 propensity-score matching based on age, sex, race, ethnicity, smoking status, and history of hypertension (Figure 9A and Supplemental Table 3). Compared with matched controls, PN patients had a higher relative risk (RR) of precursor T cell lymphoblastic leukemia/lymphoma (RR 5.33, 95% CI [2.88–9.88]), head and neck SCC (RR 4.19, 95% CI [3.22–5.45]), cervical cancer (RR 3.00, 95% CI [1.85–4.80]), breast cancer (RR 2.91, 95% CI [2.50–3.40]), bladder cancer (RR 2.83, 95% CI [1.924–4.158]), connective tissue disease (RR 2.48, 95% CI [2.15–2.87]), aortic valve calcification (RR 1.96, 95% CI [1.78–2.18]), and aortic aneurysm (RR 1.93, 95% CI [1.680–2.219]) (Figure 9B). There was no increased risk for adenoid cystic carcinoma or glioblastoma in patients with PN.

## Discussion

In this study, we investigated somatic mutational events in PN. We identified recurrent nonsynonymous somatic mutations in lesional skin, particularly in the Notch pathway. Functional validation demonstrated increased Notch signaling in PN lesional dermis, along with elevated NOTCH1 expression in fibroblasts. Furthermore, patients with PN exhibited a higher risk of several NOTCH1-associated conditions compared with matched controls. Additional findings include somatically deleted and downregulated neuronal pathway genes in PN lesional skin, particularly a 7q21 deletion observed exclusively in African American patients with PN. Of note, patients with PN had more UV-associated mutational signatures compared with AD patients, after adjusting for age and race.

Our findings highlight a role for Notch signaling in PN pathology. The Notch intracellular pathway is highly conserved and plays a critical role in cellular differentiation and tissue homeostasis (51). Abnormal Notch signaling has been implicated in various human diseases and neoplasms (52). This study identifies *NOTCH1* as the most frequently mutated gene in PN lesional skin. GSEA revealed that recurrently mutated genes in PN were associated with the Notch-mediated HES/HEY network, even after filtering out genes mutated in AD controls. *NOTCH1* mutations have been strongly associated with multiple malignancies, including cSCC. A recent meta-analysis of 83 cSCCs using multiple cancer gene discovery methods found *NOTCH1* as the top driver gene of cSCC (39). In the present study, *NOTCH1* was the most recurrently mutated gene in PN, and these mutations co-occurred with *NCOA1* mutations within the same oncogenic pathway, suggesting a proliferative role in PN lesional skin. Our comparison between PN and cSCC somatic data also identified *FAT1* as a recurrently mutated gene in PN, another driver of cSCC. Patients with PN are more likely than the general population to have coexisting health conditions, including malignancies (1, 53). In particular, previous studies found an elevated risk of SCC development in patients with PN (18). The present study also found an association between PN and several malignancies using a multicenter cohort and identified new links between PN and *NOTCH1*-associated conditions.

Beyond cellular proliferation, Notch signaling is a key driver of fibrosis in multiple organs, including renal, hepatic, pulmonary, and myocardial tissues (54–57). The profibrotic effects of NOTCH1 are likely mediated through fibroblast proliferation, myofibroblast differentiation, and immune dysregulation through TGF-β signaling (22, 58, 59). Notch activation has been observed in the lesional skin of patients with systemic sclerosis. In vitro stimulation of healthy dermal fibroblasts with a NOTCH1 ligand induced a systemic sclerosis–like phenotype, characterized by increased release of collagen and fibroblast-to-myofibroblast differentiation (60). Similarly, single-cell RNA-seq studies show an increase in myofibroblasts and cancer-associated fibroblasts (CAFs) in PN lesions (61). CAFs play a role in immune modulation and extracellular matrix remodeling (62), processes that parallel the fibroblast-driven scarring observed in PN.

Notch signaling further exacerbates fibrosis by promoting an M2-to-M1 macrophage polarization, leading to fibroblast activation and fibrocyte recruitment (63). IL-31, a well-known pruritogenic cytokine, has also been implicated in M2-to-M1 polarization (64), linking Notch dysregulation, inflammatory cytokine release, and fibrosis in PN (65, 66). Moreover, *NOTCH1* expression correlates with that of periostin (*POSTN*), a dermal extracellular matrix protein released by fibroblasts (67) that is elevated in PN; periostin expression has been shown to correlate with itch intensity (68). Recent single-cell studies in PN showed increased periostin in lesional skin and support a fibroblast-neuron axis in PN regulated by periostin (61). Given our IF analysis demonstrating increased Notch signaling in PN lesional fibroblasts, NOTCH1 hyperactivation may be a key driver of fibrosis in PN.

Due to its profibrotic and proinflammatory effects, NOTCH1 represents a promising therapeutic target in PN, and more broadly, skin fibrosis (52). Notch signaling has broad implications in human disease. As a result, several Notch-targeting therapies have been assessed and are currently under investigation in clinical trials for numerous solid tumors and blood malignancies. Future research could investigate the role of Notch inhibition, such as treatment with γ-secretase inhibitors (GSIs), to alleviate PN symptoms and lesion severity. While systemic GSIs have shown promise in preclinical models, their clinical trials were terminated due to Notch-associated adverse events, including intestinal toxicity and elevated risk of infection (69). However, nonsystemic localized Notch inhibition on the skin may offer a safer and more targeted approach, opening new avenues for treating PN and other fibrotic skin disorders.

This study also finds somatic mutations in neuronal pathways. NOTCH1 is known to inhibit neurite outgrowth in neurons, and inhibition of hyperactive Notch signaling can reverse neurogenesis and neurite outgrowth defects (70, 71). We observed recurrent nonsynonymous mutations in *MAP1B*, *TENM1*, and *CNTN2*, genes involved in neuronal projection, which were not mutated in AD and were downregulated in PN lesional skin (6), suggesting the mutations are inactivating and lead to reduced neuronal migration in PN lesional skin. Furthermore, somatic CNV analysis in PN reveals that recurrently deleted segments affect genes most primarily affect neural crest development and negative regulation of axon extension.

Previous functional studies on lesional and nonlesional PN skin biopsies show decreased intraepidermal nerve fiber density, indicating an underlying small fiber neuropathy in PN (72). However, prior studies have contested that scratching, rather than an underlying neuropathy, may be the cause of the reduced intradermal nerve fiber density, as PN lesions recovered intradermal nerve fiber density after healing (73). Our results do not support this hypothesis; rather, our mutational profiles suggest primary neuronal gene dysregulation in PN lesional skin. This is supported by association studies showing a correlation between a PN diagnosis and other systemic neuropathies (74).

Following our somatic CNV analysis, enrichment in axonal growth and guidance was largely due to a recurrent 7q21 deletion that overlaps with *SEMA3A* and related genes of the semaphorin/plexin signaling pathway. This deletion was only observed in African American patients. This is notable, considering the disproportionate burden of PN in skin of non-White patients, with African Americans having a 3.4- to 4.4-fold increased odds of developing PN compared with Whites (19, 53). In addition to CNV differences, we also observed unique high-VAF mutations affecting epithelial-mesenchymal transition in African American patients with PN, suggesting a distinct somatic evolutionary landscape in those patients. This is remarkable, given that African Americans with PN have a more fibrotic phenotype than White patients (Figure 1, B and C). Previous transcriptomic studies suggest unique patterns of immune polarization in African American patients with PN (9). Somatic CNV analysis in this study provides additional molecular evidence for the disproportionate burden of PN in African Americans.

We observed other mutations that suggest a branch of common etiology between PN and AD. The only recurrent nonsense mutation between PN and AD was in *DUOX2*, which regulates hydrogen peroxide release through NADPH oxidase (47). *DUOX2* is highly sensitive to mutation and altered protein products have been associated with elevated plasma IL-17C levels, which is characteristic of the inflammatory profiles of AD and psoriasis (47). Transcriptional and functional studies show elevated Th17 signatures in patients with PN as well, both in the skin and systemically (6, 75). However, the difference in PN is that its immunophenotype is more likely an imbalance between Th17 and Th22, with elevated levels of IL-22 (6). Interestingly, Notch signaling is shown to promote IL-22 secretion and the skewing of naive CD4^+^ T cells toward Th22 cells (76). Furthermore, NOTCH1 inhibition was shown to effectively alleviate the severity of psoriasis-like skin inflammation by regulating Th17 differentiation and function (77). Hyperactive NOTCH1 signaling can also destabilize regulatory T cells (78, 79), facilitating unrestrained Th2-driven inflammation and itch (80). The Notch pathway was highly mutated and hyperactive in PN lesional skin in this study, supporting its role in the Th17/Th22-skewed immunologic signature of PN. This is an area where precision therapeutics will make an impact, as each PN patient’s treatment can be informed by their immunologic or genomic signature.

To investigate the etiology of somatic mutational processes we observed, we performed mutational signature analysis in PN and compared it to that of AD. The relative frequency of DBS1, a highly specific signature for UV exposure with frequent tandem CC>TT mutations, was associated with PN. This was after controlling for age, race, and itch intensity. DBS1 correlated with itch intensity in both conditions. *NOTCH1* is one of the most highly mutated genes in sun-exposed versus non–sun-exposed normal human skin samples (81), and chronic UV-A exposure was shown to expand dermal fibroblasts harboring *NOTCH1* amplifications (82). Subacute skin-barrier damage may be an early event in PN patients that increases susceptibility to UV-induced DNA damage, paving the way for accumulated somatic mutations and exacerbating dysregulation in the skin microenvironment. Interestingly, we did not find DBS1 associated with age. Previous work shows a similar pattern in skin fibroblasts, where UV-associated DNA damage did not correlate with age, suggesting a proliferative origin (83).

We acknowledge certain limitations in our study. The sample size, comprising 17 PN and 10 AD cases, may have limited the detection of rare somatic events (53). Another limitation is the inability to identify somatic mutations unique to nonlesional skin, as whole blood germline controls were not collected in this study. Despite these limitations, our characterization of the somatic landscape in PN reveals insights into its pathology. Aberrant Notch signaling was identified as a potential driver in PN development, likely through profibrotic and immune deregulatory functions, with potential systemic involvement. We also provide support for the neuronal dysregulation pathophysiology of PN through recurrent loss-of-function mutations in *MAP1B*, *TENM1*, and *CNTN2*, as well as recurrent copy number deletions supported by gene expression data. Finally, our mutational signature analysis revealed a strong association between DBS1 and PN, suggesting a role for UV exposure in PN development or maintenance. Our findings represent a meaningful step toward profiling PN at the molecular level. We also add to a growing reference of somatic events in the setting of chronic inflammatory skin disease, which has the potential to transform our understanding of cutaneous biology.

## Methods

### Sex as biological variable.

Both male and female patients with PN and AD were included in this study. Sex was not used as an inclusion or exclusion criteria. Given small sample size, analyses were not stratified by sex, but the findings are expected to be relevant to both sexes.

### Sample collection.

Patients with moderate-to-severe PN, characterized by more than 20 nodules and a Worst-Itch Numeric Rating Scale (WINRS) score (84) greater than 7 out of 10, were recruited from the Johns Hopkins Itch Center. For AD controls, patients diagnosed with moderate-to-severe AD with a validated Investigator Global Assessment (vIGA) score (85) of greater than or equal to 3 and a WINRS score of more than 7 out of 10 were recruited from the Johns Hopkins Itch Center. Punch biopsies (6 mm) were collected from lesional PN or AD skin, as well as from healthy nonlesional skin within 10 cm of the nodule. Half of each biopsy was formalin-fixed, paraffin-embedded (FFPE) and the other half was stored in RNALater solution (Ambion). All patients provided informed consent, and the study was approved by the local Institutional Review Board.

### Whole-exome analysis.

Library preparation was performed with the SureSelectXT reagent kit (Agilent Technologies) before hybridization. The SureSelect XT Human All Exon V5 library was used for hybridization. An Illumina NovaSeq 6000 S4 was used for sequencing 150-bp paired-end reads. Illumina’s CASAVA (v1.8.4) was used to convert BCL files to FASTQ files. Initial quality control was performed using FastQC (v0.11.8). Trimgalore (v0.6.7) (86) was used to trim adapters, low-quality base calls, and short reads using default parameters. Following the “Best Practices” workflow suggested by the Broad Institute, BWA-mem (v0.7.17) (87) was used for alignment against the hg38 reference genome, Piccard-tools (v2.9.0) were used to mark duplicate reads, GATK (v3.8.0) IndelRealigner (88) was used to clean indel artifacts, and GATK BaseRecalibrator was used to recalibrate base quality scores and improve downstream variant calling. Samtools (v1.10) (89) and GATK were used to determine coverage at different levels of partitioning and aggregation. One AD sample was excluded due to insufficient coverage (see Results). GATK MuTect2 was used to call somatic variants. To focus on somatic events relevant to prurigo nodule development and minimize germline variant calls, MuTect2 paired mode was used, with lesional and nonlesional samples designated as the “tumor” and “normal” samples, respectively. The default parameters were used. Common germline variants and sequencing artifacts were filtered using a reference panel of normal exomes from the ExAC database; variants detected in at least 2 samples within this panel were removed. Somatic variants were then filtered using the following criteria: minimum phred quality of 20, minimum read depth of 20, and minimum variant allele frequency of 0.01. The functional effects of passed somatic SNVs and indels were then predicted using SnpEff (v5.0) (90). The R package Maftools (v2.21.05) (91) was used to summarize and visualize variant calls.

After variant calling, we considered excluding a hypermutated AD sample with 34,228 detected somatic variants after filtration. The rest of the AD samples had a median of 83.5 variants (range of 48 to 578). While this high number of variants might represent underlying germline variation or technical artifacts, it may also reflect true hypermutation related to AD. Since this study did not focus on investigating AD pathology, and the AD variants were rather primarily used to curate a gene list that is more likely to be specific to PN somatic mutagenesis, the hypermutated AD sample was not excluded.

### GSEA.

EnrichR (92) was used to perform GSEA using the following term databases: GO Biological Process, GO Cellular Component, and NCI Nature Pathways. GO terms and pathways were calculated using an α level of 0.05 after applying Benjamini-Hochberg correction and the output was visualized in R.

### Copy number analysis.

Somatic CNV was inferred using CNVkit (v0.9.4) (93), using baited genomic regions for the whole-exome target capture kit S04380110 (i.e., SureSelect Human All Exon V5). Nonlesional samples were combined into a pooled reference for CNV calling, as opposed to an individual-matched analysis, which is the recommendation of the CNVkit authors for reduced CNV noise. Recurrent CNV segments were identified using CNVRanger. This tool implements the statistical approach described by Beroukhim et al. (94), which prioritizes regions that exhibit aberrations more frequently than expected by chance. Greater weight is assigned to high-amplitude events, such as homozygous deletions or high-level copy-number gains (94).

### RNA-seq data.

In order to corroborate and contextualize the functional impact of somatic CNV calls, we utilized in-house gene expression data from lesional and nonlesional PN samples. The differential expression pipeline was described previously (6). Briefly, normalization and differential expression of RNA-seq data were carried out using the DESeq2 (95) R package, with adjustment for multiple hypothesis testing using Benjamini-Hochberg. Genes with an adjusted *P* value of less than 0.05 and log_2_(fold change) of less than 0 or greater than 0 were considered down- and upregulated, respectively. A relatively permissive absolute fold change cutoff was applied to ensure enrichment was driven by somatic CNVs.

### Mutational signature analysis.

Mutational signatures for SBSs, DBSs, and CNVs were extracted using non-negative matrix factorization, as implemented in the Sigminer R package (v2.1.7) (96, 97). CNV signatures were classified as recently described by Steele et al.; in brief, to capture biologically relevant copy number features, a CNV signature encodes the copy number profile of a sample by summing the counts of segments into a 48-dimensional vector based on total copy number, heterozygosity status, and segment size (46). The optimal number of mutational signatures was determined by analyzing the cophenetic correlation coefficient, which indicates the robustness of consensus matrix clustering. The smallest number of signatures was selected, after which the coefficient sharply decreased (98). Signatures were then compared with the curated set of COSMIC signatures v3.3 (49) using cosine similarity. Signatures with a cosine similarity of less than 0.7 to any known COSMIC signatures were considered de novo.

### Mutational burden.

We calculated mutational burden as the number of nonsynonymous mutations occurring per megabase of coding regions.

### Shannon diversity index for somatic mutations.

VAF (*v*) was calculated as the number of reads supporting the variant allele (SNV or indel) divided by the total reads supporting both the reference and variant alleles. Assuming we sequenced *n* sites, the Shannon diversity index, H, for a sample was then calculated as: 
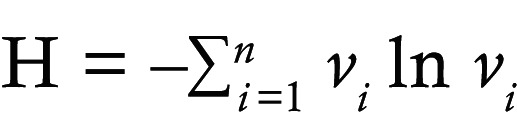


### Immunofluorescence analysis.

We selected samples from the 4 PN patients with confirmed NOTCH1 mutations. Lesional and nonlesional FFPE skin samples of 5 μm thickness were deparaffinized and subjected to heat-induced antigen retrieval using Trilogy buffer (Trilogy 920P x1, Sigma-Aldrich) and treated with DAKO protein blocking reagent (X0909, DAKO). The slides were then incubated with primary antibodies for NOTCH1 (ab52627, Abcam; 1:150) and human NICD (AF3647, R&D Systems; 1:20) at 4°C overnight, followed by reaction with conjugated secondary antibodies, DAPI intranuclear stain (62248, Thermo Fisher Scientific) and mounted with ProLong Glass Antifade Mountant (P36980, Thermo Fisher Scientific). For quantification, photomicrographs were obtained with a Leica SP8 confocal microscope (Leica Microsystems) with a 20× objective and 63× objective oil immersion lens using instrument settings. Background normalized fluorescence intensity of the antibodies in the epidermis and dermis was measured in arbitrary units (AU) using Image J software (NIH).

### GDAs.

We used DisGeNET (v7.0) (50) to identify diseases associated with *NOTCH1*. At the time of the study, DisGeNET had 1,134,942 GDAs between 21,671 genes and 30,170 diseases. Their methodology ranks diseases based on a GDA score that gives a higher weight to associations reported by several expert-curated databases and with a large number of supporting publications (50). We excluded congenital conditions (e.g., bicuspid aortic valve) and redundant terms (e.g., malignant neoplasm and breast neoplasm: only breast neoplasm was included). The 10 remaining diseases with the highest GDA scores were then selected as a priori primary outcomes in our PN cohort study.

### Cohort study.

The TriNetX Research Network is an international, federated clinical database that contained approximately 107 million patient records at the time of this study. We first identified patients diagnosed with PN who had no prior history of neoplasms. We utilized the International Classification of Diseases 10th Revision Clinical Modification (ICD-10-CM) code L28.1, which is given mostly by dermatologists and has been validated (99). Controls were identified through 1:1 propensity score matching based on age, sex, race, ethnicity, smoking status, and history of hypertension. Primary outcomes were determined through corresponding ICD-10-CM codes and association was determined through cumulative relative risk estimated using the TriNetX analytics web platform. All TriNetX analyses were completed on February 10, 2023.

### Data availability.

Sequencing data are available in the NCBI database of Genotypes and Phenotypes (dbGaP) under accession code phs004286. Values for all data points in graphs are reported in the Supporting Data Values file. Supplemental materials include additional figures and Supplemental Tables 1–4 as well as the full list of somatic variants (Supplemental Table 2).

### Statistics.

All statistical analyses and visualizations were conducted in R (version 4.2.0). Adjustment for FDR due to multiple hypothesis testing was conducted using the Benjamini-Hochberg method. An α level of 0.05 was used to denote significance. Unless otherwise specified, box-and-whiskers plots represent the following. The central box represents the interquartile range (IQR), bounded by the first quartile (Q1; 25th percentile) and third quartile (Q3; 75th percentile). The line within the box indicates the median (50th percentile). The whiskers extend to the most extreme data points within 1.5 x IQR from Q1 and Q3, respectively. Values beyond this range are plotted individually.

### Study approval.

This study was approved by the Johns Hopkins Institutional Review Board (IRB00231694).

## Author contributions

SGK, SY, A Gusev, YRS, MMK, TP, WJH, Shahin Shahsavari, and AR designed the study. VP, JD, HC, A Kollhoff, SMY, A Kambala, WA, VV, YMA, and DMG collected the data. AR, Shirin Shahsavari, A Gupta, Shahin Shahsavari, MDS, and SW analyzed the data. HC, A Kollhoff, MM, and OOO performed staining. AR, Shahin Shahsavari, and SGK wrote the first version of the manuscript. All authors reviewed and edited the manuscript. Order of authorship was determined based on overall contribution. In case of equal contribution, order was determined by mutual agreement based on project leadership and relative involvement.

## Conflict of interest

SGK is an advisory board member/consultant/speaker for Abbvie, Amgen, Arcutis Biotherapeutics, Aslan Pharmaceuticals, Bambusa Therapeutics, Bristol Myers Squibb, Cara Therapeutics, Castle Biosciences, Celldex Therapeutics, Eli Lilly, Galderma, Genzada Pharmaceuticals, Incyte Corporation, Johnson & Johnson, Leo Pharma, Novartis, Pfizer, Regeneron Pharmaceuticals, and Sanofi and has served as an investigator for Galderma, Pfizer, Incyte, Regeneron and Sanofi. SY receives research funding to his institution from Bristol-Myers Squibb and Celgene, Janssen, and Cepheid for unrelated work and has served as a consultant for Cepheid. SY owns founder’s equity in Brahm Astra Therapeutics and Digital Harmonic.

## Funding support

This work is the result of NIH funding, in whole or in part, and is subject to the NIH Public Access Policy. Through acceptance of this federal funding, the NIH has been given a right to make the work publicly available in PubMed Central.

- National Institute of Arthritis and Musculoskeletal and Skin Diseases/NIH grant K23AR077073 (to SGK).
- National Cancer Institute/NIH grant P30CA006973 (to the Sidney Kimmel Comprehensive Cancer Center Experimental and Computational Genomics Core).
- The Skin of Color Society.
- The Dermatology Foundation.

## Supplementary Material

Supplemental data

Supplemental table 2

Supporting data values

## Version 1 04/22/2026

Electronic publication
