# Supplementary material for: Somatic mutations reveal hyperactive Notch signaling in prurigo nodularis
Source: JCI Insight. 2026 Apr 22;11(8):e172371. doi: 10.1172/jci.insight.172371 (PMC13135409; doi:10.1172/jci.insight.172371)
Supplement: Supplemental data [file jciinsight-11-172371-s287.pdf]

Supplementary materials for

Somatic mutations reveal hyperactive Notch signaling in prurigo nodularis

Ahmad Rajeh[*1,2], Shahin Shahsavari[*1,2], Hannah Cornman[1,2], Alexander Kollhoff[1,2], Anuj Gupta[3], Mindy D. Szeto[1,2], Anusha Kambala[1,2], Olusola Oladipo[1,2], Varsha Parthasarathy[1,2], Junwen Deng[1,2], Melika Marani[1,2], Shirin Shahsavari[1,2], Selina M. Yossef[1,2], Vedha Vaddaraju[1,2], Yagiz M. Akiska[1,2], Davies M. Gage[1,2], Sarah Wheelan[4], Thomas Pritchard[1,2], Madan M. Kwatra[5], Yevgeniy R. Semenov[6,7], Alexander Gusev[8,9], Won Jin Ho[10], Srinivasan Yegnasubramanian[3,11], Shawn G. Kwatra[1,2]

Corresponding author email: skwatra@som.umaryland.edu

This PDF includes:

Figures S1-S3
Tables S1, S3, and S4

Other supplementary material for this manuscript:

Table S2

Figure S1. **Hypermutated PN sample.** (Related to Figure 1)

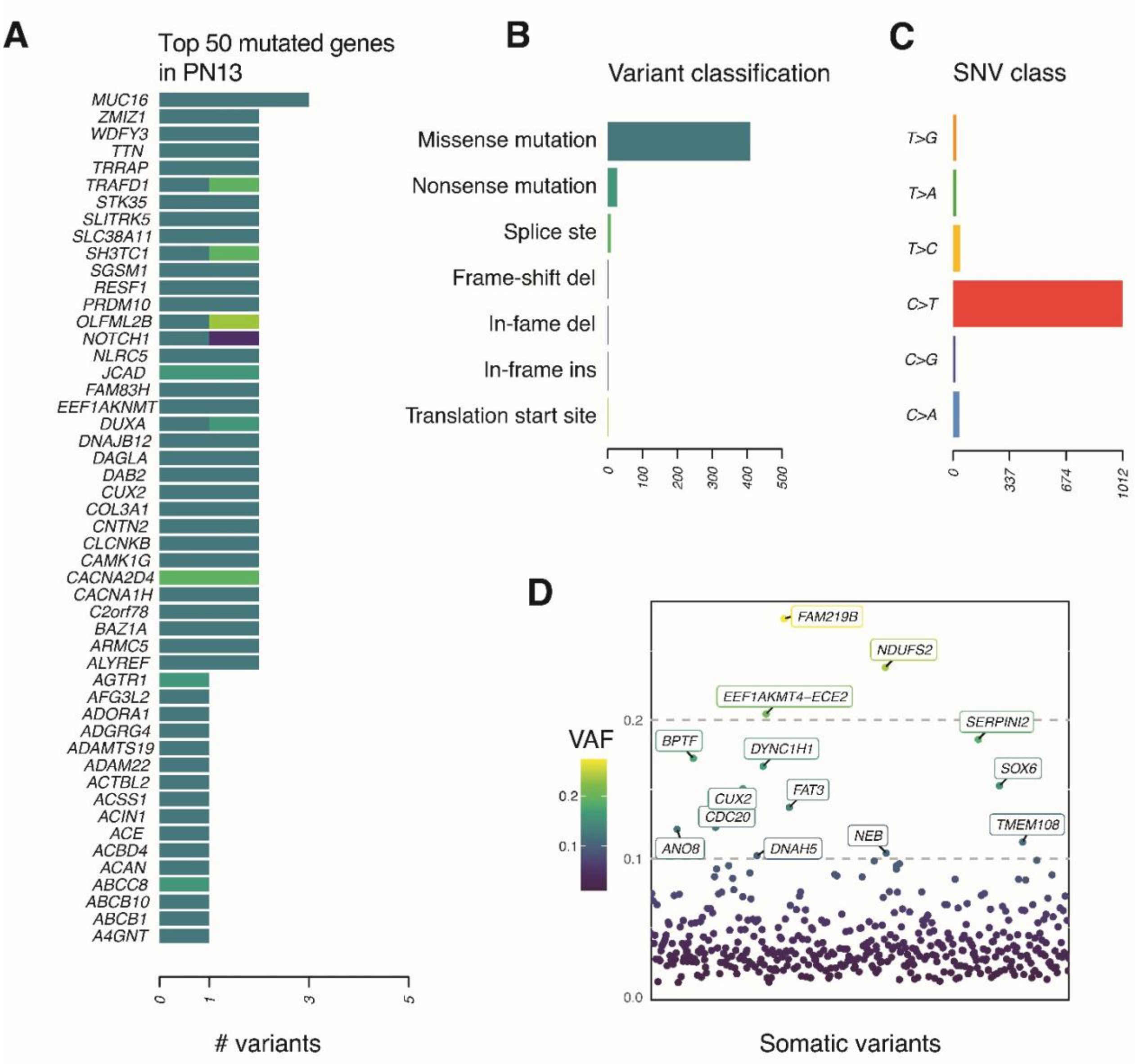

(A) List of the top 50 genes with highest nonsynonymous somatic mutations in hypermutated sample PN13. (B) Frequency of somatic mutations, classified by impact on protein function. (C) Frequency of somatic mutations, classified by single-base substitution type. (D) All nonsynonymous somatic mutations in PN13 are ordered by their genomic locations on the x-axis and the corresponding VAF is shown on the y-axis. Variants with 0.1 or higher VAF are labelled with their gene name.

Figure S2. **Gene set enrichment analyses.** (Related to Figures 4 and 5)

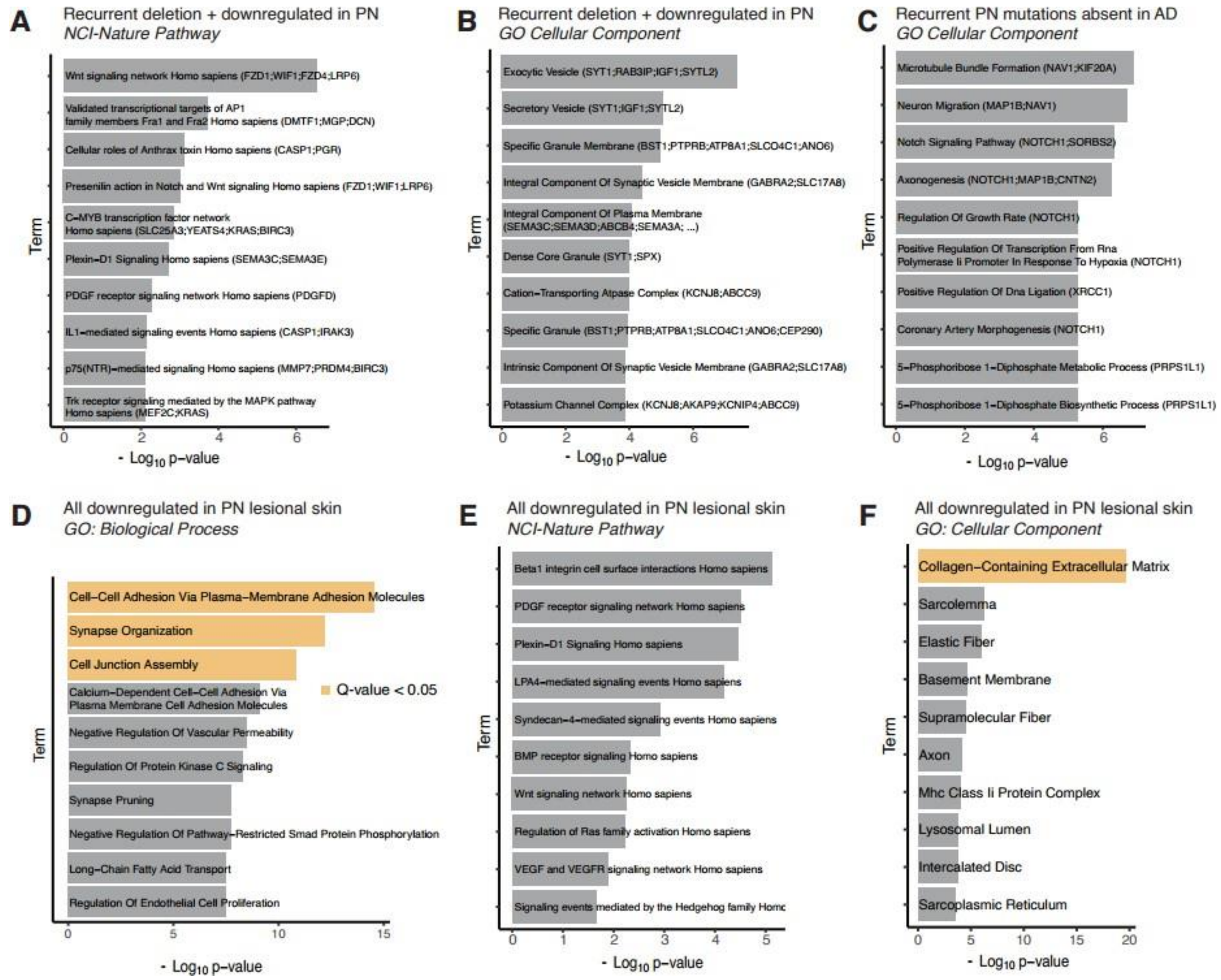

Supplemental enrichment analyses using term databases (NCI-Nature Pathway, GO: Biological Process, or GO: Cellular Component) not included in the corresponding main figures. (A-B) Term enrichment analysis of all recurrently somatically deleted and significantly downregulated genes in PN lesional skin. (C) Term enrichment analysis of 21 genes with recurrent somatic mutations in PN but none in AD. (D-F) We repeated the GO term enrichment analysis (Figure 4E) using all 3,981 genes found downregulated in PN lesional skin (without overlap with recurrent CNV information). Terms with FDR-corrected $P < 0.05$ are colored in yellow

Figure S3. Notch signaling in PN lesional skin. (Related to Figure 8)

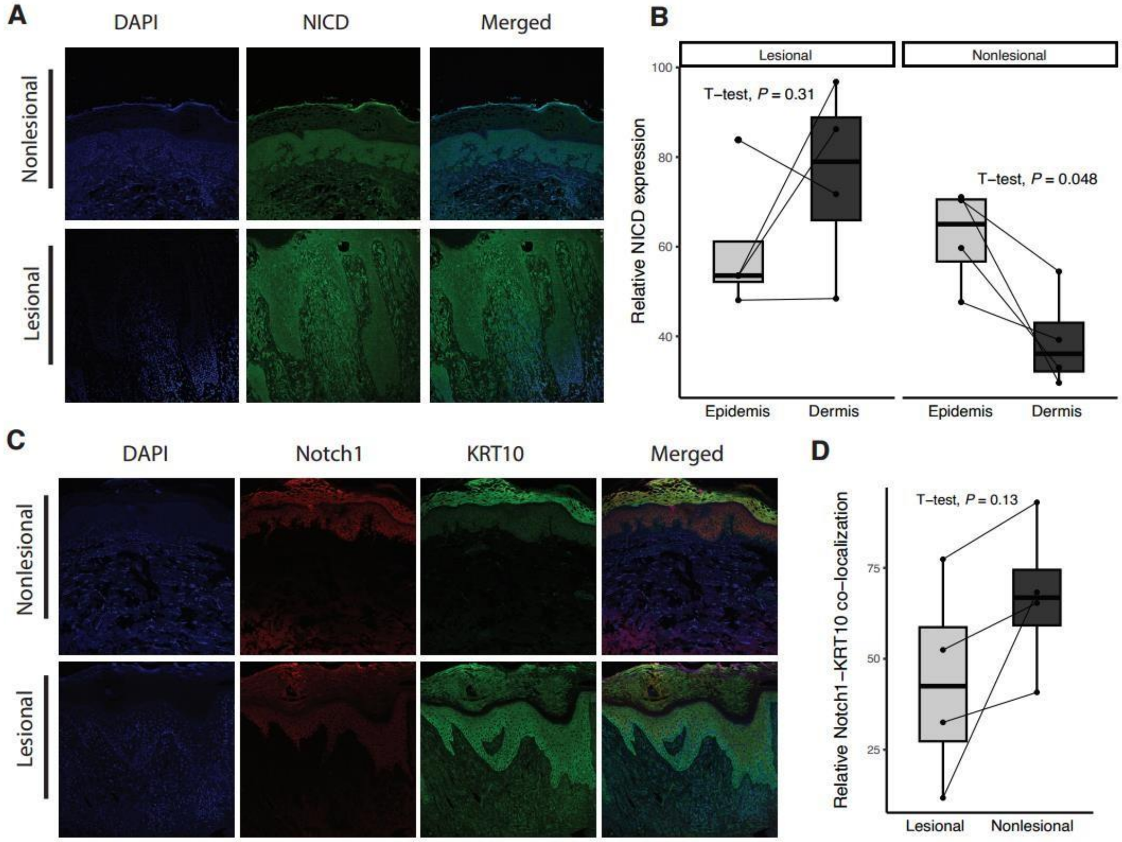

(A) IF staining of NICD in lesional and nonlesional skin sections of a PN patient with a NOTCH1 somatic mutation. Representative skin sections are shown at 40-fold magnification. NICD (green), DAPI (blue). (B) Paired boxplot showing the difference in relative expression of NICD between PN epidermis and dermis in lesional and nonlesional skin. (D) IF staining of Notch1 and KRT10 in lesional and nonlesional skin sections of a PN patient. Notch1 (red), KRT10 (green), DAPI (blue). Representative skin sections are shown at 40-fold magnification. (E) Paired boxplot showing the difference in relative co-localization of Notch1 and KRT10 between PN lesional and nonlesional skin.

Table S1. WES and alignment statistics of lesional and nonlesional samples from PN and AD patients.

Sample name	Total read pairs	Passed read pairs	Aligned read pairs	Average coverage	% base >20X
AD01_Les	46,160,417	46,142,137	46,077,328	183.67	96.4
AD01_NonLes	45,678,230	45,662,169	45,605,145	182.82	96.4
AD02_Les	58,683,142	58,658,774	58,589,249	219.94	97.1
AD02_NonLes	47,341,585	47,321,736	47,199,560	185.11	96.8
AD03_Les	41,011,419	40,998,518	40,945,801	165.46	95.7
AD03_NonLes	49,878,240	49,860,942	49,778,484	192.24	96.9
AD04_Les	50,742,258	50,709,341	50,635,699	204.51	53.7
AD04_NonLes	45,693,665	45,675,292	45,593,139	179.16	96.1
AD05_Les	41,445,120	41,429,614	41,370,519	163.26	96.2
AD05_NonLes	49,800,687	49,783,616	49,686,145	192	95.5
AD06_Les	41,386,664	41,369,475	41,299,752	163.8	95.4
AD06_NonLes	65,787,405	65,761,670	65,668,988	241.97	97.3
AD07_Les*	46,401,360	46,384,217	46,327,118	183.71	91.1
AD07_NonLes*	72,787,080	72,701,107	72,546,883	53.18	38
AD08_Les	45,164,463	45,151,660	45,092,200	174.15	96.5
AD08_NonLes	45,390,243	45,376,554	45,290,384	178.13	95.9
AD09_Les	45,260,181	45,245,888	45,178,713	179.05	96.2
AD09_NonLes	42,098,717	42,081,084	42,006,100	164.85	96.1
AD10_Les	40,730,421	40,718,688	40,111,319	156.56	94.7
AD10_NonLes	46,809,990	46,796,804	46,112,521	178.93	96.5
PN01_Les	43,381,478	43,366,279	43,312,068	166.27	95.9
PN01_NonLes	46,282,752	46,267,595	46,204,071	184.69	96.3
PN02_Les	54,976,043	54,956,221	54,899,077	203.57	97
PN02_NonLes	43,732,978	43,716,597	43,676,125	170.4	96.1
PN03_Les	56,006,884	55,984,451	55,838,863	212.06	96.9
PN03_NonLes	52,560,601	52,543,300	52,429,199	206.13	96.7
PN04_Les	44,112,770	44,098,137	44,028,956	165.99	96.4
PN04_NonLes	47,608,236	47,590,535	47,498,737	188.24	96.4
PN05_Les	56,556,972	56,534,406	56,460,825	214.81	97.1
PN05_NonLes	61,487,706	61,461,160	61,303,243	233.73	97.5
PN06_Les	55,350,460	55,327,426	55,223,154	207.25	97.2
PN06_NonLes	56,776,503	56,755,447	56,685,661	221.79	97.4
PN07_Les	56,030,559	56,012,202	55,884,880	210.31	96.9
PN07_NonLes	53,717,595	53,695,884	53,572,557	208.09	97
PN08_Les	41,541,426	41,526,931	41,479,114	161.69	87
PN08_NonLes	48,556,258	48,539,888	48,468,857	187.16	93.8
PN09_Les	44,216,679	44,201,428	44,137,089	166.75	96
PN09_NonLes	49,515,506	49,492,487	49,406,572	203.44	70.7
PN10_Les	48,842,653	48,825,855	48,769,389	185.38	95.4

PN10_NonLes	45,896,235	45,862,121	45,740,188	171.24	97.6
PN11_Les	43,736,958	43,718,000	43,658,231	166.16	87.8
PN11_NonLes	64,810,969	64,770,853	64,674,866	236.42	96.2
PN12_Les	46,788,922	46,762,189	46,695,344	182.81	64.4
PN12_NonLes	47,106,451	47,085,153	46,951,000	183.59	96
PN13_Les	61,319,095	61,292,730	61,178,061	231.72	97.2
PN13_NonLes	70,449,117	70,423,391	70,251,859	268.64	97.7
PN14_Les	47,644,189	47,624,440	47,572,723	177.18	96.3
PN14_NonLes	50,869,288	50,850,639	50,762,462	201.05	96.6
PN15_Les	45,439,842	45,423,436	45,362,594	175.81	96.2
PN15_NonLes	56,946,952	56,924,773	56,798,890	219.91	97.1
PN16_Les	45,850,373	45,834,554	45,795,353	178.79	95.9
PN16_NonLes	47,672,931	47,656,841	47,529,912	186.9	96.7
PN17_Les	42,740,629	42,726,532	42,663,696	166.86	96.1
PN17_NonLes	43,243,709	43,224,747	43,165,870	169.62	96.2

*Patient excluded due to low percent of high coverage basepairs in the nonlesional sample.

Table S2. All somatic variants identified in lesional PN or AD samples compared to matched nonlesional samples.

Table_S2.xlsx

Table S3. Dictionary of TCGA cancer symbols. (Related to Figure 5)

Symbol	Cancer name
ACC	Adrenocortical carcinoma
BLCA	Bladder urothelial carcinoma
BRCA	Breast invasive carcinoma
CESC	Cervical squamous cell carcinoma and endocervical adenocarcinoma
CHOL	Cholangiocarcinoma
COAD	Colon adenocarcinoma
DLBC	Lymphoid neoplasm diffuse large b-cell lymphoma
ESCA	Esophageal carcinoma
GBM	Glioblastoma multiforme
HNSC	Head and neck squamous cell carcinoma
KICH	Kidney chromophobe
KIRC	Kidney renal clear cell carcinoma
KIRP	Kidney renal papillary cell carcinoma
LAML	Acute myeloid leukemia
LGG	Brain lower grade glioma
LIHC	Liver hepatocellular carcinoma
LUAD	Lung adenocarcinoma
LUSC	Lung squamous cell carcinoma
MESO	Mesothelioma
OV	Ovarian serous cystadenocarcinoma
PAAD	Pancreatic adenocarcinoma
PCPG	Pheochromocytoma and paraganglioma
PRAD	Prostate adenocarcinoma
READ	Rectum adenocarcinoma
SARC	Sarcoma
SKCM	Skin cutaneous melanoma
STAD	Stomach adenocarcinoma
TGCT	Testicular germ cell tumors
THCA	Thyroid carcinoma
THYM	Thymoma
UCEC	Uterine corpus endometrial carcinoma
UCS	Uterine carcinosarcoma
UVM	Uveal melanoma

Table S4. Basic characteristics of the PN and matched control patients in the TriNetX Research Network cohort study. (Related to Figure 8). Patients with prior neoplasms were excluded.

Cohort	Characteristic	Mean ± SD	Patients	% of cohort	P-value
**PN**	Age at Index	50.2 +/- 18.2	42,397	100%	0.985
**Controls**		50.2 +/- 18.2	42,397	100%	
**PN**	White		22,986	54.20%	0.995
**Controls**			22,985	54.20%	
**PN**	American Indian or Alaska Native		192	0.50%	0.878
**Controls**			189	0.40%	
**PN**	Female		25,665	60.50%	0.961
**Controls**			25,672	60.60%	
**PN**	Native Hawaiian or Other Pacific Islander		60	0.10%	0.928
**Controls**			61	0.10%	
**PN**	Unknown Ethnicity		8,385	19.80%	0.979
**Controls**			8,382	19.80%	
**PN**	Not Hispanic or Latino		30,596	72.20%	0.994
**Controls**			30,597	72.20%	
**PN**	Hispanic or Latino		3,416	8.10%	0.98
**Controls**			3,418	8.10%	
**PN**	Black or African American		9,259	21.80%	0.993
**Controls**			9,258	21.80%	
**PN**	Male		16,725	39.40%	0.961
**Controls**			16,718	39.40%	
**PN**	Unknown Race		8,247	19.50%	1
**Controls**			8,247	19.50%	
**PN**	Asian		1,653	3.90%	0.943
**Controls**			1,657	3.90%	
**PN**	Nicotine dependence, cigarettes		942	2.20%	0.981
**Controls**			941	2.20%	
**PN**	Hypertensive diseases		11,289	26.60%	0.994
**Controls**			11,288	26.60%	
